# Exploring Yeast Diversity to Produce Lipid-Based Biofuels from Agro-Forestry and Industrial Organic Residues

**DOI:** 10.3390/jof8070687

**Published:** 2022-06-29

**Authors:** Marta N. Mota, Paula Múgica, Isabel Sá-Correia

**Affiliations:** 1 iBB—Institute for Bioengineering and Biosciences, Instituto Superior Técnico, Universidade de Lisboa, Av. Rovisco Pais, 1, 1049-001 Lisbon, Portugal; 2 Department of Bioengineering, Instituto Superior Técnico, Universidade de Lisboa, Av. Rovisco Pais, 1, 1049-001 Lisbon, Portugal; 3 i4HB—Institute for Health and Bioeconomy, Instituto Superior Técnico, Universidade de Lisboa, Av. Rovisco Pais, 1, 1049-001 Lisbon, Portugal; 4 BIOREF—Collaborative Laboratory for Biorefineries, Rua da Amieira, Apartado 1089, São Mamede de Infesta, 4465-901 Matosinhos, Portugal

**Keywords:** oleaginous yeasts, yeast diversity, biofuels, lignocellulosic biomass, industrial organic residues, microbial lipids, microbial oils, yeast biorefineries, circular bioeconomy

## Abstract

Exploration of yeast diversity for the sustainable production of biofuels, in particular biodiesel, is gaining momentum in recent years. However, sustainable, and economically viable bioprocesses require yeast strains exhibiting: (i) high tolerance to multiple bioprocess-related stresses, including the various chemical inhibitors present in hydrolysates from lignocellulosic biomass and residues; (ii) the ability to efficiently consume all the major carbon sources present; (iii) the capacity to produce lipids with adequate composition in high yields. More than 160 non-conventional (non-*Saccharomyces*) yeast species are described as oleaginous, but only a smaller group are relatively well characterised, including *Lipomyces starkeyi*, *Yarrowia lipolytica*, *Rhodotorula toruloides*, *Rhodotorula glutinis*, *Cutaneotrichosporon* *oleaginosus* and *Cutaneotrichosporon cutaneum*. This article provides an overview of lipid production by oleaginous yeasts focusing on yeast diversity, metabolism, and other microbiological issues related to the toxicity and tolerance to multiple challenging stresses limiting bioprocess performance. This is essential knowledge to better understand and guide the rational improvement of yeast performance either by genetic manipulation or by exploring yeast physiology and optimal process conditions. Examples gathered from the literature showing the potential of different oleaginous yeasts/process conditions to produce oils for biodiesel from agro-forestry and industrial organic residues are provided.

## 1. Introduction

The sustainable production and use of renewable fuels to enable the transition to a low-carbon and more sustainable economy have been promoted in recent years in response to the global climate crisis and the growing energy needs [1,2]. Liquid biofuels play a central role in that transition, in particular biodiesel which is considered an ideal candidate for the replacement of petroleum-derived diesel due to its high cetane number and flash point and the possibility to be used in any compression-ignition engine without the need for modification [3,4]. Biodiesel results from the reaction of one triacylglycerol molecule (consisting of three long-chain fatty acids attached to glycerol) with three alcohol molecules (usually methanol or ethanol) to produce three biodiesel molecules, i.e., methyl esters or ethyl esters, and one glycerol molecule [5,6]. Biodiesel is currently mainly produced through the transesterification of oils, in particular vegetable oils (including edible oils) or animal fats [7]. However, the use of these sources is of concern as they can compete with the food oil market and implicate increased deforestation and biodiversity loss by intensifying the use of land for cultivation [8]. To overcome these disadvantages, microbial lipids have gained attention in recent years. Besides not competing with food, their production is not susceptible to seasonal changes, the growth of microorganisms is much faster and microbial oils production can be optimised and controlled in bioreactors, with their composition being very similar to that of vegetable oils [9,10,11]. Among the best microbial candidates capable of producing lipids in high concentrations and with appropriate characteristics are oleaginous yeasts, described as those capable of accumulating more than 20% of cell dry weight in lipids [11,12]. In addition to the aforementioned advantages, oleaginous yeasts also have the potential to metabolise diverse carbon sources of difficult catabolism, can exhibit high tolerance to a wider range of relevant bioprocess-associated stresses and have an unusual and specialised metabolism producing a wide and diverse repertoire of lipids, proteins and metabolites with high commercialisation potential [13,14,15,16,17]. Although there are over 160 yeast species described as oleaginous, only a small set of these species are relatively well characterised, including *Lipomyces starkeyi, Yarrowia lipolytica*, *Rhodotorula glutinis*, *Rhodosporidium/Rhodotorula toruloides*, *Cutaneotrichosporon oleaginosus* (previously classified as *Cryptococcus curvatus* or *Trichosporon oleaginosus*) and *Cutaneotrichosporon cutaneum* (formerly known as *Trichosporon cutaneum*) [18]. These non-*Saccharomyces* yeast species are referred to as non-conventional yeasts. However, based on their biotechnological interest and current intense research, it is anticipated that they will soon, if not already, stop being considered as such.

The economic viability of industrial lipid production bioprocesses depends on the performance of the yeast strain under optimised conditions and the efficient use of low-cost renewable raw materials, such as lignocellulosic biomasses [19,20]. Extensive screenings of yeast strains of various species available in culture collections and of new strains isolated for this purpose were carried out. Studies to improve selected yeast strains for better performance using lignocellulosic biomass hydrolysates or other interesting feedstocks with emphasis on diverse wastes were also performed. However, the use of genetic engineering techniques requires the availability of appropriate tools to be applied to the non-conventional yeast species of interest. If they are not available or not efficient enough, the exploration of other more traditional genetic improvement techniques is a possible approach. The optimisation of conditions for the production process (e.g., pH, temperature, medium and other culture conditions, type of reactors) is equally required.

This literature review presents recent results that support the idea that there is a huge interest and potential in several oleaginous yeast species/strains to generate oils for biodiesel production, in particular when produced from lignocellulosic biomasses from forestry and agriculture and industrial organic by-products/residues/wastes. The challenges faced by yeasts when cultivated in these feedstocks are discussed, including the catabolism of different carbon sources (C-sources), lipid biosynthetic pathways, and tolerance to the toxic compounds present and to other relevant stresses. Results gathered from recent literature concerning the production of yeast oils from different feedstocks, from a circular bio-economy perspective, are also provided.

## 2. Diversity of Oleaginous Yeasts

**Figure 1:**
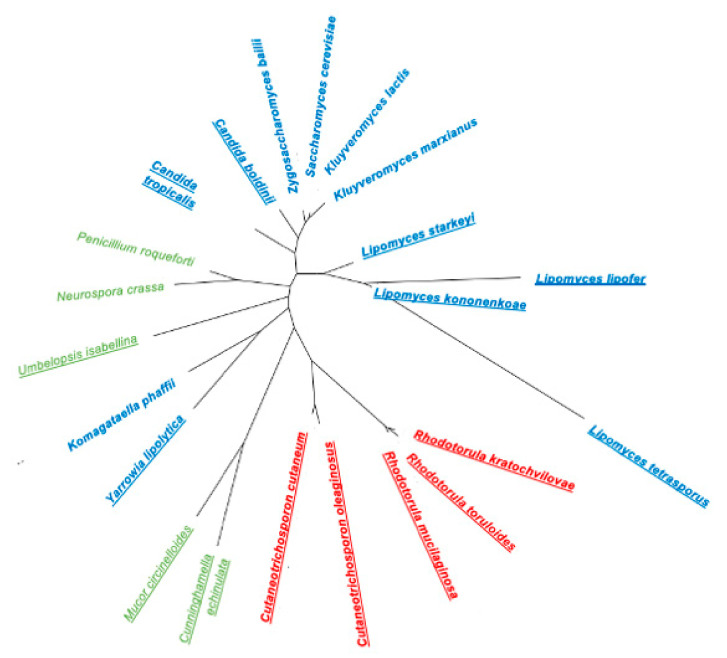
Phylogenetic tree of biotechnologically relevant fungi (yeasts and filamentous fungi), highlighting the diversity of oleaginous yeasts. The tree was constructed using the maximum-likelihood method based on the alignment of the small subunit (18S) ribosomal DNA sequence. The sequences used were obtained from NCBI database. The underlined species were described as oleaginous. Ascomycete yeasts are shown in blue, basidiomycetous yeasts are in red and filamentous fungi are in green.

Oleaginous yeasts belong to the two phyla within the kingdom Fungi, Ascomycota and Basidiomycota. The diversity of oleaginous yeasts is observed in the phylogenetic tree prepared for biotechnologically relevant yeasts (Figure 1).

In the phylum Ascomycota, yeasts from the genera *Lipomyces* and *Candida*, and the species *Yarrowia lipolytica* were extensively studied due to their intrinsic lipid production potential [18,21,22,23]. There are sixteen species of the genus *Lipomyces,* with the *Lipomyces starkeyi* and *Lipomyces tetrasporus* species being isolated worldwide [24]. Besides being present in the soil, some species were also isolated from insect-associated habitats such as frass, decaying cactus tissues and tree fluxes [24]. *L. starkeyi* is the species of the *Lipomyces* genus with a larger number of published studies due to its high lipid productivity [11,25]. *Y. lipolytica* is a dimorphic yeast usually found in environments containing hydrophobic substrates, rich in alkanes and fats. It can be isolated from cheese, yoghurt, kefir, soy sauce, meat and shrimp salads [26]. The genome sequences of strains *Y. lipolytica* Po1f, commonly used for metabolic engineering, and the reference strain CLIB122, are available [27] and there are several synthetic biology tools for *Y. lipolytica* genetic manipulation [28]. They include DNA assembly techniques for synthetic biology, DNA parts for expression cassette construction, genome-editing techniques, and computational tools [28]. Regarding the *Candida* genus, the species *Candida boidinii*, *Candida utilis*, and *Candida tropicalis* were reported as oleaginous [22,29]. *C. boidinii* strains were isolated from natural environments (soil, seawater, sap fluxes of many sugar-rich tree species) or samples associated with human activities (wine fermentations or olive manufacturing), suggesting a biotechnological potential [30].

Regarding the phylum Basidiomycota, *Rhodotorula (Rhodosporidium*) *toruloides*, *Cryptococcus curvatus* and species of the genus *Trichosporon* are interesting lipid producers. *Rhodotorula* species are present in several habitats such as bark-beetles, tree exudates, plants and vegetables, soil, fresh water, coastal sediments and marine environments but were also isolated from clinical samples [31]. These yeasts, known as red yeasts, exhibit a red colour due to the production of carotenoids and can exist both in the yeast or in mycelial forms [18]. Remarkably, the species *R. toruloides* presents a huge potential as a workhorse for biotechnological applications [32]. One of the most extensively studied *Cryptococcus* species, *C. curvatus* (now, *Cutaneotrichosporon oleaginosus*), can accumulate up to 73% dry cell weight (DCW) in lipids [33]. It is distributed in nature and can be isolated from foodstuffs (raw milk, lettuce) and marine sediments [11]. The majority of *Trichosporon* strains were isolated from soil and milk whey samples [30] but some strains were also isolated from immunocompromised hosts. The potential pathogenicity may limit the use of this and other species for industrial applications.

The ability of oleaginous yeasts to grow in low-cost substrates can be related to the habitats from where these oleaginous yeasts are isolated and a considerable number of oleaginous yeasts are primarily found in soils, in particular *L.* *starkeyi* and *L. tetrasporus,* and some *Cryptococcus* and *Trichosporon* species [18]. The type of soil enhances the ability of the yeasts present there to consume a wide variety of substrates, such as lignocellulosic biomasses [24]. *L. starkeyi, T. cutaneum* and some species of *Cryptococcus* are tolerant to the major inhibitors present in lignocellulosic biomass hydrolysates, including acetic acid, furfural, and 5-hydroxymethylfurfural (HMF) [34,35,36,37,38]. Additionally, *R. toruloides* and *R. mucilaginosa* are able to consume the acid sugar galacturonic acid from hydrolysates from sugar beet pulp, a pectin-rich residue [39,40]. Xylose, the second most abundant sugar in lignocellulosic biomass hydrolysates, is used as a carbon source (C-source) for growth by all the aforementioned genus/species. Even though many *Yarrowia* isolates readily consume xylose [41], *Y. lipolytica* Po1-derived strains require genetic modifications to be able to use this pentose as a carbon source [42]. Glycerol can also be used as a C-source for lipid production by *C. boidinii*, *C. curvatus*, *L. starkeyi*, *R. toruloides* and *Y. lipolytica* [25,43,44]. Therefore, crude glycerol, a byproduct of the biodiesel industry is potentially an interesting substrate for oil production by all these yeast species, especially for those also capable of catabolising and tolerating methanol, a major contaminant in crude glycerol [45].

## 3. Oleaginous Yeasts for Sustainable Biodiesel Production

### 3.1. Biosynthesis of Yeast Oils

Biodiesel is produced through the transesterification of oils involving the conversion of triacylglycerols (TAGs) to fatty acid methyl (or ethyl) esters (FAMEs) [46]. The structure, including the chain length of the fatty acids produced, can vary and determines the quality of the biofuel. Oleaginous yeasts accumulate non-polar lipids, such as steryl esters (SEs) and triacylglycerols (TAGs), in particular mystiric acid [C14:0], palmitic acid [C16:0], stearic acid [C18:0], oleic acid [C18:1], linoleic acid [C18:2], and linolenic acid [C18:3] [19]. Fatty acid-lipid profiles can vary depending on culture conditions and yeast species/strains. Remarkably, the FAMEs derived from oleaginous yeast have similar properties to more traditional sources derived from food crops such as rapeseed, palm or sunflower oils [25,46,47]. Oleaginous yeasts can produce different oils, with oleic acid (18:1) being the oil that is produced in higher titers. For example, in *L. starkeyi* NBRC 10381, oleic acid content represents nearly 74% of the total produced oils [48]. Since oleic acid is the lipid that best meets the criteria to obtain biodiesel with the best properties [49,50], yeasts are highly interesting cell factories for sustainable biodiesel production.

### 3.2. Triacylglycerol (TAG) Metabolism in Yeasts

**Figure 2:**
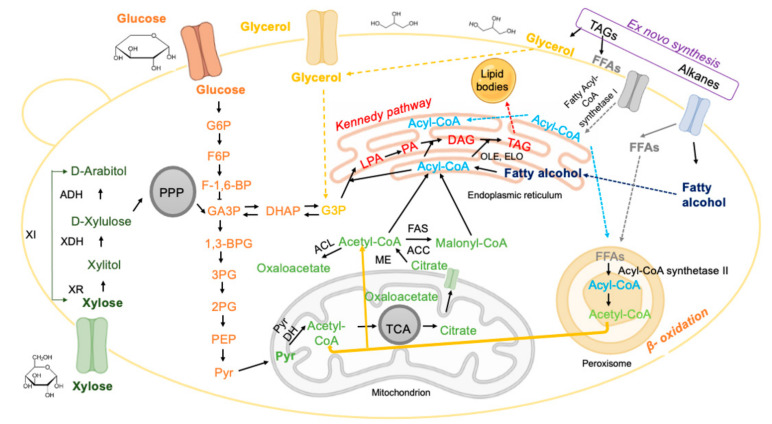
Lipid biosynthesis pathways in oleaginous yeasts. The pathways involved in lipid synthesis (de novo and ex novo synthesis) are summarised in this figure. The explanation of each step of the pathway is described in the main text. Abbreviations: ACC, Acetyl-CoA carboxylase; ACL, ATP-citrate lyase; ADH, alcohol dehydrogenase; 1,3-BPG, 1,3-bisphosphoglycerate; DAG, diacylglycerol; DHAP, dihydroxyacetone phosphate; ELO, Elongase; ER, endoplasmic reticulum; F6P, fructose-6-phosphate; F-1,6-BP, Fructose 1,6-bisphosphate; FAS, fatty acid synthase; FFA, free fatty acids; G6P, glucose-6-phosphate; G3P, glycerol-3-phosphate; GA3P, glyceraldehyde-3-phosphate; LPA, lysophosphatidic acid; OLE, desaturase; 2PG, 2-phosphoglycerate; 3PG, 3-phosphoglycerate; PA, phosphatidic acid; PEP, phosphoenolpyruvate; PPP, pentose phosphate pathway; Pyr, pyruvate; Pyr DH, pyruvate dehydrogenase; TAG, triacylglycerol.

Triacylglycerols (TAGs) can be synthesised and accumulated by: (i) de novo synthesis, when the precursors of fatty acid biosynthesis are produced from different carbon sources (e.g., sugars, weak acids, glycerol), or (ii) ex novo synthesis, based on the fatty acids present in the culture medium (Figure 2).

#### 3.2.1. De Novo Synthesis

The de novo synthesis pathway of TAGs is triggered by the limitation of the nitrogen source when the carbon source is in excess, i.e., in a culture medium with a high C/N ratio [19,42], as detailed in Section 6.2. The biochemical reactions involved in the de novo synthesis of lipids are schematised in Figure 2. Under nitrogen-limiting conditions, adenosine monophosphate deaminase (AMPD) is activated and catalyses the hydrolysis of adenosine monophosphate (AMP) to inosine monophosphate (IMP) and ammonia, thereby providing nitrogen to the cell [51]. At low AMP concentrations, isocitrate dehydrogenase activity decreases [52] and the tricarboxylic acid cycle (TCA) becomes dysregulated, leading to isocitrate accumulation. Through the action of the enzyme aconitase, isocitrate and citrate levels balance and citrate is transported from the mitochondria to the cytosol via malate/citrate antiport [53]. Once in the cytosol, citrate is converted into acetyl-CoA and oxaloacetate by ATP citrate lyase (ACL), a key enzyme during lipogenesis. The next steps include (i) the conversion of oxaloacetate to malate, and (ii) the cleavage of malate into pyruvate and NADPH. The pyruvate enters the pyruvate dehydrogenase complex (PDH cycle) where pyruvate is decarboxylated, producing acetyl-CoA, the key molecule for lipid production, as well as NADH and carbon dioxide. Fatty acid synthesis begins with cytosolic acetyl-CoA being condensed into malonyl-CoA, in a reaction catalysed by acetyl-CoA carboxylase. Acetyl-CoA and malonyl-CoA are condensed to acyl-CoA by the fatty acid synthase complex (FAS). NADPH is used as a reducing cofactor by fatty acid synthase and two molecules of NADPH are used in each step of acyl-CoA chain elongation. The most common chain length of naturally synthesised acyl-CoAs has 16 or 18 carbon atoms. The C16:0 and C18:0 molecules are routed to the endoplasmic reticulum (ER) in order to proceed to the elongation and desaturation steps [54]. The synthesis of TAGs is carried out via the Kennedy pathway, starting with glycerol-3-phosphate (G3P) from glycolysis and acyl-CoA [55]. Two fatty acids (FA) are added to the glycerol structure by two acetyltransferases. Glycerol-3-phosphate acyltransferase (GPAT) converts G3P to lysophosphatidic acid (LPA) [56] and lysophosphatidic acid is acetylated by LPA acyltransferase. The latter reaction produces phosphatidic acid (PA), which is dephosphorylated to diacylglycerol (DAG) in a reaction performed by phosphatidic acid phosphatase (PAP) [57]. The last step comprises the acylation of TAGs, at the sn-3 position, either by an acyl-CoA-dependent or an acyl-CoA-independent reaction, to form TAGs that are stored in the form of lipid droplets [11,23].

#### 3.2.2. Ex Novo Synthesis

In the ex novo pathway, hydrophobic substrates such as esters, TAGs, alkanes, etc., present in the culture medium are hydrolysed and transported to the intracellular space by active transport systems. There are two alternatives regarding the fate of the released fatty acids: they can be stored in lipid droplets, or they can be used for growth after the beta-oxidation of fatty acids. In both alternatives, the following step consists of the conversion of free fatty acids into acyl-CoA, a reaction catalysed by acyl-CoA synthetase [19]. Acyl-CoA can be esterified with glycerol, producing reserve and structural lipids [22]. Microbial lipids produced via the ex-novo pathway contain lower amounts of TAGs and higher amounts of free fatty acids compared to lipids produced via the de novo process [22].

## 4. Production of Yeast Oils from Lignocellulosic Biomass Hydrolysates: Inherent Challenges

The results of the extensive screenings of several yeast species/strains for the utilisation of different types of lignocellulosic biomasses to produce lipids are summarised in Table 1. Corn-derived biomasses show promising results with the highest lipid content described for a corn cob hydrolysate, reaching values of about 73% [58] and the highest lipid concentration values for the bioconversion of corn straw (23.3 g/L in a bioreactor, produced by *R. toruloides* DSMZ 4444). The highest lipid concentration (39.6 g/L) was obtained from Jerusalem artichoke extract hydrolysate using *R. toruloides* Y4 [59]. These results reinforce the idea of the potential of *R. toruloides* for lipid production from different feedstocks.

The negative impact of growth inhibitors that arise from the pretreatment of lignocellulosic substrates or the challenges registered in the catabolism of several C-sources by several oleaginous yeast species are discussed below (Section 4.1, Section 4.2 and Section 4.3).

### 4.1. Inhibition of Yeast Growth and Metabolism by Toxic Compounds Generated during Pretreatment

Lignocellulosic biomass is the largest renewable resource in the world. It is composed of complex carbohydrate polymers consisting of cellulose, hemicellulose, lignin and, depending on the biomass, a more or less residual part that includes pectin, proteins, extracts and ash [78,79]. Due to the recalcitrant nature of this biomass to deconstruction, a combination of enzymatic and thermochemical pretreatment processes is required to release the sugar components that can be converted into different value-added bioproducts by yeasts or other microorganisms [11,20]. Lignocellulosic biomass pre-treatments depend on the nature, chemical composition and structure of the biomass (hardwood, softwood or herbaceous) [80]. The nature and concentration of the by-products generated depend on the pre-treatment but may include furans and aldehydes, 2-furaldehyde (furfural) and 5-(hydroxymethyl)furfural (HMF), aromatic compounds (vanillin, syringaldehyde and 4-hydroxybenzoic acid) and weak acids (acetic, formic and levulinic acids) [80,81]. Since hemicellulose and lignin are acetylated [20,82], acetic acid is frequently present in lignocellulosic biomass hydrolysates at concentrations that can reach toxic values [83,84,85,86] being considered one of the major inhibitory compounds in lignocellulosic biomass hydrolysates. However, several strategies were developed to minimise the toxic effect of acetic acid, either by implementing a pretreatment that tackles the deacetylation and mechanical refining (DMR) or by the conversion of acetate directly into lipids [87] or co-products [88]. For its importance, the role of acetic acid in this context is detailed in Section 4.3. The concentrations of the main compounds present in lignocellulosic hydrolysates obtained after different biomasses pre-treatments were compiled [20,89]. Depending on the concentrations attained, they can seriously compromise yeast growth and bioconversion performance [80].

The furan-derived compounds, 2-furaldehyde (furfural) and 5-(hydroxymethyl)furfural (HMF) are formed during biomass pretreatment by the dehydration of pentoses and hexoses, respectively, and affect the activity of key enzymes of cellular metabolisms, such as glycolytic enzymes [90]. Furthermore, due to the action of the reactive aldehyde groups of furfural and HMF [34,37,91], reactive oxygen species (ROS) accumulate and may oxidise proteins, lipids and nucleic acids, affecting the corresponding cellular structures and leading to increased mutagenesis, protein denaturation, and biomembrane damage [90,92]. For this reason, the intrinsic tolerance of oleaginous yeasts to furan-derived compounds is also considered critical.

For detoxification of furfural and HMF, yeasts use reductases and dehydrogenases, that reduce or oxidise them to less toxic alcohols (furfuryl alcohol and 2,5-bis-hydroxymethylfuran) or acids (furoic acid and 2,5-furan-dicarboxylic acid) [35,93,94]. Furoic acid showed a lower toxic effect in *Trichosporon fermentans* when compared with furfural or furfuryl alcohol, inhibiting sugar utilisation rate less markedly [35]. However, it is important to note that tolerance to these furan compounds, as to any other toxicant, is strain-dependent [91,94]. Most *Rhodotorula* species are able to tolerate furfural concentrations up to 0.5 g/L. Two notable examples are the *R. graminis* strain UCDFST 04-862, which tolerates more than 0.5 g/L of HMF [91] or the *R. pacifica* strain INDKK, which is able to survive to 0.5 g/L of HMF and 2 g/L of furfural [95]. The tolerance of the *R. graminis* strain to HMF was increased using adaptive laboratory evolution experiments (ALE) by incubation in a corn maceration liquor medium supplemented with HMF (0.4%) for 7 days [96]. A *Pichia kudriavzevii* strain, isolated from soil, was found to be able to tolerate exceptional levels of HMF, up to 7 g/L [97]. There is a wide range of robust non-conventional yeasts with a natural tolerance to furfural and HMF while maintaining the ability to accumulate lipids as shown in Table 2. *Trichosporon cutaneum* 2.1374 is a good example as it is able to grow and produce lipids in media containing up to 1 g/L furfural or 2 g/L HMF more efficiently when compared to other species under the same conditions [34]. Although there are no in-depth studies available on the tolerance mechanisms active in oleaginous yeast species to these furan derivatives [98], the knowledge obtained in model yeasts is useful to guide strategies for increasing their tolerance to these and other stresses associated to related bioprocesses [99,100]. The development of more tolerant strains is discussed in Section 7.

### 4.2. Limitations to the Efficient and Complete Use of All the C-Sources Present, in Particular Xylose

The complete and efficient use of the sugars and other potential C-sources present in lignocellulosic biomass hydrolysates or in hydrolysates from any other feedstocks is essential to make their conversion economically viable. In lignocellulosic biomass hydrolysates, the main challenge is the bioconversion of xylose, which, in general, is the second most abundant sugar [101,102]. In the case of pectin-rich biomasses and residues, the acid sugar galacturonic acid is another highly challenging C-source for catabolisation by yeasts but can be efficiently catabolised by some oleaginous species [39,40]. Native xylose metabolism is not common in the Saccharomycotina but fairly common throughout the non-conventional yeast species [103]. Among other factors, carbon catabolite repression (CCR) represses xylose utilisation if glucose is present, in particular, the transport of sugars into the cell. This species-specific regulation leads to the sequential, rather than simultaneous, use of these C-sources as a result of preferential use of glucose, or another repressing carbon source, over others also present [104,105,106]. For this reason, CCR negatively affects the performance of biotechnological processes, since it prolongs the production time and, consequently, increases the inherent costs. This means that CCR is a very important regulatory mechanism when the use of mixtures of different C-sources is envisaged, as it is the case of hydrolysates from biomass or organic by-products or residues/wastes. The systematic study of lipid accumulation and production kinetics in a variety of oleaginous ascomycetous and basidiomycetous yeast strains grown on glucose and xylose, followed by the use of the selected strains for the bioconversion of wheat straw hydrolysate, pointed out as promising strains of the species *L. starkeyi*, *R. glutinis*, *Rhodotorula babjevae* and *R. toruloides* [107].

**Figure 3:**
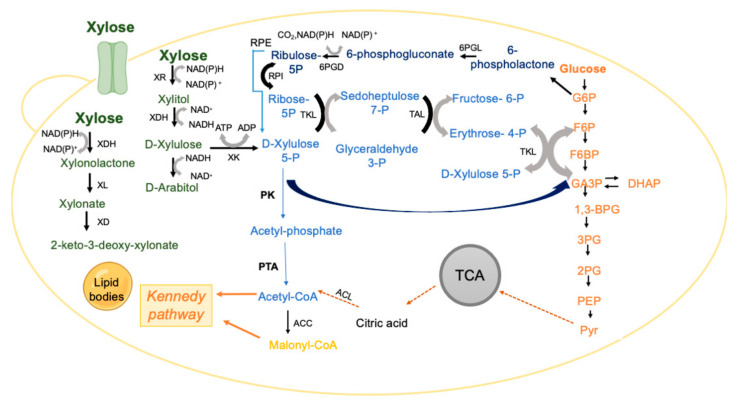
Metabolic pathways of xylose assimilation in yeast. The oxidative pentose phosphate pathway is highlighted in dark blue, and the non-oxidative pentose pathway is shown in light blue. Abbreviations: ACC, Acetyl-CoA carboxylase; ACL, ATP-citrate lyase; ADH, alcohol dehydrogenase; ADP, adenosine diphosphate; ATP, adenosine triphosphate;1,3-BPG, 1,3-bisphosphoglycerate; CO_2_, carbon dioxide; DAG, diacylglycerol; DHAP, dihydroxyacetone phosphate; F6P, fructose-6-phosphate; F-1,6-BP, Fructose 1,6-bisphosphate; FAS, fatty acid synthase; FFA, free fatty acids; G6P, glucose-6-phosphate; G3P, glycerol-3-phosphate; GA3P, glyceraldehyde-3-phosphate; G6PD, glucose 6-phosphate dehydrogenase; 6PGL, 6-phosphogluconolactonase; 6PGD, 6-phosphogluconate dehydrogenase; RPI, ribose-5-phosphate isomerase; R LPA, lysophosphatidic acid; NAD, nicotinamide adenine dinucleotide; 2PG, 2-phosphoglycerate; 3PG, 3-phosphoglycerate; PA, phosphatidic acid; PEP, phosphoenolpyruvate; PK, phosphoketolase; PPP, pentose phosphate pathway; PTA, phosphotransacetylase; Pyr, pyruvate; Pyr DH, pyruvate dehydrogenase; RPE, ribulose 5-phosphate 3-epimerase; RPI, ribose-5-phosphate isomerase; TAG, triacylglycerol; TAL, transaldolase; TCA, tricarboxylic acid cycle; TKL, transketolase; XD, xylonate dehydratase; XDH, xylitol dehydrogenase; XI, xylose isomerase; XK, xylulose kinase; XR, xylose reductase.

One of the limiting steps during xylose conversion, justified by CCR, is the xylose transport into the cell since xylose transporters are less efficient than those responsible for glucose transport, mainly due to low selectivity and/or affinity towards xylose [108,109]. Therefore, the identification of xylose transporters in *L. starkeyi*, *R. toruloides* and *Y. lipolytica* using molecular, bio-informatic, enzymatic, and transcriptomic analyses constitutes a starting point for the development of engineered strains for lipid production from xylose-rich substrates, [108,110,111,112,113]. The uptake of xylose into the yeast cell is followed by the activity of the xylose oxidoreductive pathway [102,114,115] (Figure 3). Briefly, xylose is reduced to xylitol, a reaction catalyzed by xylose reductase (XR) that uses NADH or NADPH as a cofactor [103,116]. The enzyme xylitol dehydrogenase (XDH) converts xylitol to D-xylulose by reducing NAD^+^ to NADH [117]. These two steps cause a redox imbalance that can be another limiting step in many yeasts. D-xylulose is then phosphorylated to xylulose-5-phosphate (X5P) by xylulose kinase (XK) [118]. The latter metabolite enters the phosphoketolase (PK) pathway, or the non-oxidative pentose phosphate pathway [102,103].

*R. toruloides* and *L. starkeyi* species can actively assimilate xylose [119,120]. *R. toruloides* tends to accumulate arabitol [114,121], produced through D-xylulose, in a reaction catalysed by the enzyme alcohol dehydrogenase (ADH) with consequent NAD^+^ production, which could be coupled to the reaction catalysed by XDH (Figure 3). Thus, these two reactions can contribute to the redox balance during xylose assimilation, with arabitol accumulation increasing under unbalanced conditions [121]. In addition, some *Rhodotorula* species, such as *R. graminis*, *R. glutinis* or *R. toruloides* metabolise X5P that can be transformed into glyceraldehyde-3-phosphate and acetyl-phosphate via the phosphoketolase (PK) pathway [122,123]. The PK pathway is more efficient if carbon economy is considered, as acetyl-phosphate can bypass pyruvate decarboxylation [102]. *L. starkeyi* was also shown to produce arabitol (about 1 g/L) when grown on corn stover hydrolysate, suggesting that it might also possess the arabitol production pathway [124].

Due to the limited genetic tools for both *Rhodosporidium* sp./*Rhodotorula* sp. and *Lipomyces* sp., genetic and metabolic engineering studies are scarce and have not yet allowed the detailed clarification of their xylose assimilation pathway. *Y. lipolytica* is known to possess in its genome genes encoding xylose reductase, xylitol dehydrogenase and xylulose kinase, but they are not sufficiently expressed to allow the efficient utilisation of xylose [125]. Thus, several genetic engineering strategies were explored to improve xylose assimilation in this species (detailed in Section 7.3).

### 4.3. The Dual Role of Acetic Acid as a Metabolism Inhibitor and C-Source

Acetate concentrations as high as 15 g/L can be found in lignocellulosic hydrolysates [126] and, depending on medium pH, significantly inhibit yeast growth and metabolism compromising sugar consumption rate and lipid yield [19,85,86]. At a pH below the pKa of this weak acid, (4.75 at 25 °C), acetic acid is essentially in the toxic undissociated form and enters the cell through the lipid bilayer of the plasma membrane by simple diffusion. In the cytosol, at a pH close to neutrality, acetic acid dissociates and the release of a proton (H^+^) leads to a decrease in intracellular pH and acetate accumulation [85,86]. Increased oxidative stress and turgor and inhibition of yeast growth and metabolism are among the detrimental effects of acetic acid toxicity [85,86]. Studies dedicated to the mechanisms of adaptation and tolerance to acetic acid in yeasts, in particular at the genome-scale in *S. cerevisiae*, are available in the literature and in several review papers [83,84,85,86,127,128,129]. Changes that occur in the molecular composition, structure and physical properties of the plasma membrane and cell wall are among the adaptive responses to this weak acid [130,131,132,133]. Because of such modifications, the permeability of the cell envelope in adapted cells is reduced, and so is the rate of passive diffusion of the acid form into the cell. This response, coordinated with the action of plasma membrane efflux pumps, such as Aqr1, Tpo2 and Tpo3, is reported to catalyse the active expulsion of intracellular acetate out of the cell, leading to the decrease in the internal concentration of the acid, and, consequently, its toxicity [134,135]. Regarding cell wall remodelling in response to acetic acid stress, a recent study reports that an adaptive response towards a more rigid and robust cell wall is also critical for acetic acid tolerance [130]. This response limits the futile cycle associated with the re-entry of the toxic acid form after the active expulsion of acetate from the cell interior [130]. The crosstalk between the ergosterol content of yeast plasma membrane and cell wall biophysical properties, involving the plasma membrane ABC transporter Pdr18, described as a determinant of acetic acid tolerance due to its involvement in ergosterol transport at the plasma membrane level, was also demonstrated [131]. Considering the high importance that acetic acid toxicity has in the performance of yeasts, in particular oleaginous yeasts, the exploration of yeast biodiversity and a better understanding of the molecular targets and pathways behind the increase in yeast efficiency and robustness under stress imposed by acetic acid is essential to the productivity and economic sustainability of lignocellulosic biorefineries [136].

Although toxic, acetic acid can also be an interesting C-source for oleaginous yeasts. However, it is important to use a cultivation medium pH leading to lower acetic acid toxicity and acetic acid concentrations below the threshold for each yeast strain tolerance [39,137,138]. Acetate can be converted into acetyl-CoA, which is a precursor for lipid biosynthesis, so most yeasts capable of assimilating acetic acid are oleaginous. In oleaginous fungi, a considerable percentage of acetate is directed to lipid biosynthesis in the presence of glucose and xylose, as in the case of lignocellulosic biomass hydrolysates [139]. Furthermore, acetate can be directly converted to acetyl-CoA in the cytosol by acetyl-CoA synthetase (ACS) and immediately used for fatty acid biosynthesis without the involvement of complex and energy-consuming metabolic and mitochondrial transport processes [139].

In the presence of glucose, acetic acid assimilation can be repressed in yeasts such as *S. cerevisiae*, *Candida utilis*, *Torulaspora delbruecki* and *Dekkera anomala* [98]. However, in other yeasts, acetate can be simultaneously catabolised, as in the case of *Zygosaccharomyces bailii* [140] and *R. toruloides* [39,141]. Several studies indicate that there is an increase in lipid production when the co-consumption of sugars and acetic acid occurs [19,126,142]. When acetic acid is co-consumed with xylose, sugar assimilation can be facilitated as well as lipid accumulation [19,126,142]. For example, the presence of acetate and its co-metabolism with glucose-enhanced lipid content to levels close to 70% in the presence of 7.2 g/L acetate, indicates that the excess acetate is used as building blocks in lipid biosynthesis by *R. toruloides* [143]. A *Cryptococcus curvatus* strain was able to simultaneously consume mixtures of (i) acetate and glucose, (ii) acetate and xylose, and (iii) acetate in rich corn hydrolysates, and produce lipids. Furthermore, the partial replacement of glucose by acetic acid in the same amount resulted in higher lipid concentration (6.8 g/L in medium with 30g/L glucose and 10 g/L acetic acid compared with 6.0 g/L lipid concentration, obtained in the medium with 40 g/L glucose) [142]. In the case of *Trichosporon cutaneum* 2.1374, this strain was able to slowly metabolise acetic acid simultaneously with glucose or xylose, and lipid productivity was also higher in acetic acid supplemented medium. Acetic acid can be used either as the sole C-source or as a C-source in the second stage of two-stage fermentation (Table 3). In the latter case, the increase in acetate concentration leads to a higher C/N ratio, suitable for lipid production (see Section 6.2) [143]. For example, with 20 g/L of acetic acid, *R. toruloides* AS 2.1389 cells accumulated 48.2% in lipids, whereas with 4 g/L of this acid, the lipid content was reduced to approximately one-third of that value [141]. Acetic acid can also exert a beneficial effect on lipid production when it is present in a mixture of volatile fatty acids (VFAs), a topic discussed in Section 5.2.

## 5. Production of Yeast Oils from Organic Industrial Byproducts/Wastes/Residues

### 5.1. From Crude Glycerol, a Biodiesel Production Byproduct

**Figure 4:**
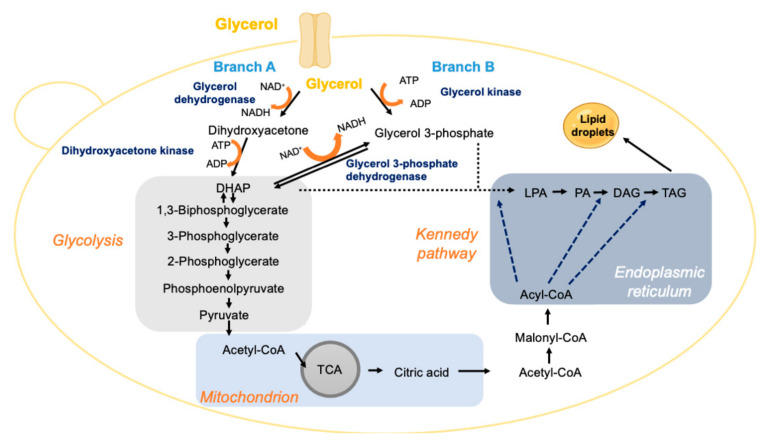
Metabolic pathways involved in glycerol catabolisation for the production of lipids. Abbreviations: ADP, adenosine diphosphate; ATP, adenosine triphosphate; DAG, diacylglycerol; LPA, lysophosphatidic acid; NAD, nicotinamide adenine dinucleotide; PA, phosphatidic acid: TAG, triacylglycerol.

Crude glycerol is a byproduct of biodiesel manufacturing that can be used as feedstock for the production of yeast oils (Table 4). Crude glycerol produced in the biodiesel industry is composed of 70–80% glycerol that can be used as a C-source for lipid production by suitable oleaginous yeasts (Figure 4). However, although crude glycerol composition varies depending on the industrial process, it is contaminated with alcohols (mainly methanol), catalysts, dissolved salts, and water [146,147].

The use of glycerol as a carbon source by oleaginous species, as is the case for *C. boidinii*, *C. curvatus*, *L. starkeyi*, *R. toruloides* and *Y. lipolytica* [25,43,44], occurs through the phosphorylation and oxidative pathways. For *S. cerevisiae* and *Y. lipolytica*, the active transport mechanism primarily uses glycerol/H+ antiporters [44]. Regarding the oxidative pathway (Branch A, Figure 4), the first step consists of the oxidation of glycerol to dihydroxyacetone through FAD/NAD dehydrogenase. Dihydroxyacetone is phosphorylated by a dihydroxyacetone kinase, producing dihydroxyacetone phosphate [6]. The other alternative reaction involves the enzyme 3-phosphoglycerate dehydrogenase (NAD-dependent), which catalyses the conversion of glycerol-3-phosphate into dihydroxyacetone phosphate in the mitochondria [44]. Once in the cytosol, dihydroxyacetone enters the glycolytic pathway (Branch B, Figure 4) and follows the TCA and Kennedy pathways (Figure 4). Considering the phosphorylation pathway, glycerol is phosphorylated by glycerol kinase, generating glycerol-3-phosphate that can enter directly into the Kennedy pathway for lipid production.

Since most biodiesel manufacturers utilise high methanol-to-oil molar ratios, methanol is a major contaminant of crude glycerol residues and methanol toxicity affects the performance of oleaginous yeasts [45]. Although part of this alcohol can be removed by thermal treatment, the residual methanol concentration may be toxic to yeast cells and limit bioprocess productivity [161]. Lipid production by *R. toruloides* 32489 using crude glycerol supplemented with increasing methanol concentrations (from 2 to 20 g/L) was inversely proportional to methanol concentration: at 20 g/L, biomass, lipid content and lipid production dropped by 6.6%, 11.9% and 17.7%, respectively, compared with pure glycerol [155]. However, when mixed with other impurities present in crude glycerol substrates such as esters (e.g., methyl and sodium oleate), salts and soap, the negative effects of methanol can, apparently, be alleviated [155]. Moreover, methanol can be useful in avoiding bacterial contamination of non-sterilised crude glycerol used for lipid production [150,152].

Methanol can also be used as a C-source by yeasts but no study addressing methanol consumption and lipid production from crude glycerol could be found in the literature. Recent reports on the exploitation of different methodologies to optimise the utilisation of methanol as a C-source by methylotrophic and non-methylotrophic yeasts were published [161,162,163,164]. According to a metabolomics study, the methylotrophic yeast *Ogataea methanolica* responds to the presence/absence of methanol and also to its concentration [164]. The native capacity of S. cerevisiae for methylotrophy was examined as the first step towards the unraveling of methylotrophy in the model yeast [163]. Synthetic methylotrophy constitutes a challenging alternative that can be implemented in non-methylotrophic oleaginous hosts to increase the feasibility of bioprocesses that use crude glycerol as a substrate.

### 5.2. From Volatile Fatty Acids (VFAs), Intermediate Compounds from Anaerobic Digestion of Organic Wastes

Volatile fatty acids (VFAs) are intermediate compounds obtained from anaerobic digestion of organic wastes, for example, food wastes. VFAs are produced after the hydrolytic and acidogenic phases of anaerobic digestion. The most common VFAs are acetic (C2), propionic (C3), butyric (C4), isovaleric, valeric (C5) and caproic (C6) acids [165], and their ratio depends on the experimental conditions, substrate composition and the microorganisms present in the anaerobic digestion system [166]. VFAs are considered a promising alternative feedstock for lipid production by oleaginous yeasts in a circular bio-economy context [19,145,167,168]. The main studies available in the literature using a mixture of VFAs as carbon sources for lipid production are summarised in Table 5. When *Y. lipolytica* was grown on a mixture of VFAs, acetic acid was found to play a key role in the consumption of longer-chain VFAs (C5 and C6), increasing the availability of the C-sources suitable for lipid production [169]. Additionally, a higher proportion of acetic acid in the VFA mixture of acetic acid:propionic acid:butyric acid (in ratios of 8:1:1) led to higher lipid accumulation, lipid concentration and productivity when compared to lower proportions of acetic acid in the VFA mixture (e.g., acetic acid:propionic acid:butyric acid in ratios of 4:3:3 or 6:1:3) [170].

### 5.3. From Combinations of Residues/Wastes

Different combinations of crude glycerol and other carbon sources present in lignocellulosic biomasses or other organic industrial residues were also explored [43,151,157,175,176,177]. The addition of cellulosic hydrolysates to crude glycerol improved the lipid production rate of *Rhodotorula* species. The mixing of crude glycerol with 10% hydrolysate from wheat straw subjected to the acid-based steam explosion (composition: 2.6 g/L xylose, 0.6 g/L glucose and 0.8 g/L acetic acid) led to an enhanced lipid production rate and the reduction of the time for consumption of all the available carbon sources of *R. toruloides* and *R. glutinis.* The valorisation of crude glycerol and sunflower meal (SFM) resulting from biodiesel production plants was also examined using *R. toruloides*, *L. starkeyi* and *C. curvatus*. Among the aforementioned species, the lipid profile of *R. toruloides* oils was the closest to the palm oil used for biodiesel [157]. The strategy of fed-batch cultivation, using sugarcane top hydrolysate as a substrate in the first stage and crude glycerol in the second stage was found to have a strong influence on biomass and lipid production in *Rhodosporidiobolus fluvialis* DMKU–SP314 [175,177]. Genetically engineered microorganisms are also an alternative to enhance lipid production using low-cost residual substrates. A good example is the genetically manipulated *Y. lipolytica* JMY4086, which is able to successfully catabolise crude glycerol and molasses, producing lipids [176].

For feedstocks with a low C/N ratio, such as in wastewater sludge, the addition of crude glycerol as a promising C-source for lipid production can be considered an interesting strategy [178]. For example, the use of a combination of municipal sludge fortified with crude glycerol, allows *Y. lipolytica* SKY7 to produce higher concentrations of biomass and lipids when compared with unsupplemented crude glycerol, leading to the valorisation of these two byproducts/wastes [148].

A first study focusing on the combination of pumpkin peels with syrup from candied fruits processing was recently published, demonstrating that these wastes were sufficient to support yeast growth and enhance lipid accumulation in *Rhodosporidiobolus azoricus* and *Cutaneotrichosporon oleaginosum* [179].

## 6. Effect of Process Conditions in the Production of Yeast Oils

Several physiological and environmental factors affect the growth, lipid accumulation and lipid profile of oleaginous yeasts. These include yeast species/strain, growth phase, culture medium components (e.g., carbon (C)-source, nitrogen (N)-source, molar C/N ratio), and other macronutrients (e.g., phosphorus and sulphur) as well as micronutrients (trace metals in minimal media) and undefined micronutrients in complex media, and other cultivation conditions (e.g., inoculum size and physiological state, pH, temperature, dissolved oxygen (DO) level, type of bioreactor(s), cultivation time). The optimisation of these factors is essential to achieve high productivity and minimise production costs.

### 6.1. Nitrogen (N) Source

Lipid production is influenced by the nature and concentration of the carbon and nitrogen sources used by oleaginous yeasts. The influence of the C-source was discussed above (Section 4.2, Section 4.3, and Section 5.1, Section 5.2 and Section 5.3). Concerning the N-source, both organic (yeast extract, peptone or urea) and inorganic (ammonium chloride, ammonium sulphate and sodium nitrate, or a mixture of both) nitrogen were tested [180,181,182]. Since yeast extract is an expensive medium component, its replacement by other low-cost organic nitrogen sources (e.g., corn steep liquor, monosodium glutamate, soybean powder or urea) was explored [71]. The results revealed that when half of the yeast extract was replaced by urea, a significant decrease in lipid concentration occurred (from 6.6 g/L to 4.9 g/L) [71]. This could be the result of the alkalinisation of the culture medium by the ammonium ion resulting from urea hydrolysis, known to trigger *L. starkeyi* cell death [124]. However, the use of urea or even ammonia as major nitrogen sources is well documented for *Y. lipolytica* [183,184]. Inorganic sources, such as ammonia, are preferred in industrial processes due to the lower cost. However, if organic and inorganic nitrogen sources are compared, organic nitrogen sources are more favorable for lipid accumulation, as described for *R. toruloides*, with an oil content of 50% when grown on organic nitrogen compared to 18% when inorganic nitrogen was used [185]. This result may also be related to the likely presence of some nutrients such as amino acids and vitamins in organic sources that may enhance cell growth and lipid accumulation [186].

### 6.2. Carbon-to-Nitrogen (C/N) Ratio

Lipid production by oleaginous microorganisms requires a medium in which there is an excess of carbon and a limited amount of other nutrients such as phosphorus, sulphur or nitrogen [9]. The excess carbon is redirected to lipid synthesis, rather than to cell proliferation [187]. As previously described in Section 3.2.1, it is considered that nitrogen depletion triggers the activation of adenosine monophosphate deaminase and catalyses the conversion of AMP to inosine 5’-monophosphate and ammonium, initiating the TAG synthesis reaction [188]. Therefore, the carbon-to-nitrogen (C/N) ratio is critical during lipid biosynthesis. C/N ratios suitable for lipid production range from 50 to 150 [189]. However, it is essential to establish a suitable C/N ratio that favors lipid accumulation without compromising cell growth in the medium. A reported exception to the referred production profile is the case of *Cryptococcus terricolus* which accumulates lipids when there is still nitrogen in the culture medium [190].

Specific examples supporting the general conclusions stated above follow. When *R. toruloides* CCT 0783 was grown with four different C/N ratios (60, 80, 100 and 120) and three different C-sources (glycerol, acetic acid or xylose), the highest lipid yields in acetic acid and xylose were for a C/N ratio of 120 (0.6 g/g and 0.53 g/g, respectively) [191]. However, for glycerol, a C/N ratio as high as 120 caused a marked decrease in specific growth rate and lipid yield [191]. In a study using *Trichosporon dermatis* 32903 that compares the influence on lipid production of C/N ratios from 30 to 130, the ratio of 110 led to the highest lipid production (16.33 g/L) [75]. For *R. taiwanensis* AM2352, the highest amount of lipids accumulated was at a C/N ratio of 30 [69]. Collectively, these results emphasise the importance of choosing an appropriate C/N ratio according to the carbon source, the selected strain and other cultivation conditions.

In addition to the initial C/N ratio, the amount of nitrogen per se should also be considered [124]. In other words, increasing the amount of the initial carbon source may not always be sufficient to increase lipid production if cells only start accumulating lipids when nitrogen concentration is low enough [124]. As a strategy to achieve high lipid concentrations without compromising biomass production, two-stage batch processes were used in which cell proliferation occurs first, in a rich medium, and lipid accumulation occurs later, under nitrogen-limiting conditions [19,60]. Under such conditions, lipid production by *L. starkeyi* NRRL Y-1388 increased by 78% [192]. When *L. tetrasporus* Y-11562, *L. kononenkoae* Y-7042 and *R. toruloides* Y-1091 were used and a C/N 60 was present in the first phase and a C/N ~500 in the second phase, lipid productivity was three to seven times higher than was possible during the first growth phase [60]. Since the aim of the second phase is to produce lipids and not biomass that accumulates during the first phase, it is possible to use higher concentrations of C-sources that also act as growth inhibitors. This is a strategy used for yeast species with poor growth on acetic acid [145,193], or to enhance lipid yield in species capable of using acetic acid efficiently [141,170].

### 6.3. Dissolved Oxygen (DO) Concentration

Dissolved oxygen (DO) concentration affects both lipid accumulation and composition, although the results reported in the literature vary with the yeast species. Typically, oleaginous yeasts require oxygen for rapid growth; in bioreactors, agitation increases nutrient availability while maintaining uniformity of cell distribution in the medium [58]. However, in general, high aeration levels lead to a decrease in the lipid content but the optimum aeration level depends on the yeast strain [124,194,195,196]. For example, for DO levels of 25% and 60%, the higher lipid accumulation by *R. glutinis* was at the lower DO level while higher DO levels favor biomass production [196]. However, not all yeast species are equally affected by the DO concentration concerning lipid accumulation [197]. For example, *Rhododosporium azoricum’s* production of lipids was found to be more prone to low DO concentrations than *Trichosporon oleaginous* [197]. Finally, it is important to note that fatty acid desaturases use oxygen as a substrate to catalyse the unsaturation reaction [124], but there is no unequivocal association between higher saturation and dissolved oxygen levels [196,198].

### 6.4. Temperature and pH

The cultivation temperature also influences the composition of yeast oils and their degree of saturation and the optimum temperatures for which biomass production is favoured, may not be optimal for lipid accumulation [71,194,199]. Additionally, the activity of yeast desaturases is temperature-dependent, these enzymes being more stable at low temperatures, namely the Δ12-desaturase [188,200], thus, the saturation degree of yeast oils is also temperature-dependent [138,201].

The optimum pH for lipid production should be selected for specific substrates and strains [11,22,187]. When glucose is the main carbon source, acidic conditions, mainly in the range of pH 5 to 6, are employed in lipid production [137]. Remarkably, *L. starkeyi* is capable of growing and producing lipids in media with a very low pH, around 3.0, likely due to their intracellular buffering capacity [63,184]. In the case of *R. mucilaginosa* and *R. toruloides*, the presence of acetic acid in the hydrolysates (30–40 mM) adjusted to pH 5.0 did not compromise the rapid and full utilisation of D-glucose, D-galactose and acetic acid [39]. Nevertheless, at pH 3.5, yeast growth was fully abrogated [39].

Regarding the use of VFAs as a carbon source, there are two different perspectives. Some authors consider that slightly acidic conditions (pH 5.6–7) are beneficial compared to alkaline conditions, mainly for low concentrations of VFAs. Other authors report that alkaline pHs can be advantageous since they alleviate the toxic effect of the high content of the weak acids present in VFA mixtures, enhancing lipid production [137]. In the case of cultivation media with high content of VFAs, an initial pH of 8 was found to be the optimal pH condition for lipid production by *Y. lipolytica* [137].

### 6.5. Effect of the Inoculum

The size and physiological state of the inoculum are critical to the performance of stress-associated bioprocess, as is the case for most of the bioconversions of lignocellulosic biomass and other organic residues/wastes by yeasts. Additionally, the inoculum size influences biomass production, lipid titer and lipid content [202,203,204,205]. It is expected that an increase in the inoculum size (frequently associated with the initial culture OD_600nm_) may lead to the increase in the concentration of viable producing cells capable of initiating growth under stress conditions, in particular under the toxic effect of chemicals present in lignocellulosic biomass hydrolysates or in any other organic residues [65]. A higher active cell fraction elevates the probability of the cell population resuming growth after sudden exposure to a stressful environment and exhibiting an increased C-source consumption rate [202]. For example, the negative impact on *R. toruloides* performance of inhibitory concentrations of acetic acid present in sugar beet pulp (SBP) hydrolysates was negligible when higher concentrations of inoculum were used [39]. A similar increase in process performance concerning the consumption rate of a mixture of xylose and glucose was reported for *L. starkeyi* when the inoculum size was increased [202]. An inoculum ratio of 10% (*v*/*v*) was considered ideal for maximum biomass and lipid production, and lipid content by *Phenoliferia glacialis* (syn, *Rhodotorula glacialis*) DBVPG4875 [203] and *Rhodotorula kratochvilovae* (syn, *Rhodosporidium kratochvilovae*) SY89 [204] but other values were found depending on the specific bioprocess conditions [119,205].

## 7. Strategies to Develop Superior Strains for the Production of Oils from Residual Feedstocks

### 7.1. Exploring Available Bioinformatics Tools

For guiding the development of superior yeasts by genetic and genome engineering, in particular for non-conventional yeasts, several web database resources provide a wealth of functional and transcription regulation information for the analysis of gene expression datasets. This is the case of the *Saccharomyces* Genome Database (SGD) (https://www.yeastgenome.org/, accessed on 7 June 2022), the major community resource for gene, genomic and protein information in yeast and the YeastIP database that compiles nucleotide sequences of the most common markers used for yeast taxonomy and phylogeny, allowing identification, taxonomy and phylogeny of yeasts species [206]. The YEASTRACT+ database and information system, a tool for the analysis of transcription regulatory associations in *Saccharomyces cerevisiae,* currently includes the N.C.Yeastract database (Non-Conventional Yeastract; http://yeastract-plus.org/ncyeastract/, accessed on 7 June 2022) [207,208]. N.C.Yeastract allows the (i) inference of orthologous genes, (ii) search for putative TF binding sites, and (iii) inter-species comparison of transcription regulatory networks and prediction of TF-regulated networks based on documented regulatory associations available in YEASTRACT + for well-studied species, especially *S. cerevisiae* [208]. For example, the prediction of the Haa1 regulon in *R. toruloides* (RtHaa1) in response to acetic acid stress was possible using YEASTRACT+ [209]. The outcome of such analysis can have an impact on the optimisation of *R. toruloides* robustness for the bioconversion of lignocellulosic and pectin-rich residue hydrolysates [209] given that the transcription regulator Haa1 is a major determinant of acetic and formic acids tolerance in yeasts [85].

Other in silico approaches are available to facilitate the development of superior yeasts. Genome-scale metabolic models available for several oleaginous yeasts such as *L. starkeyi* [210,211], *R. toruloides* [212], *T. oleaginosus* (*Cutaneotrichosporon oleaginosus*) [213], and *Y. lipolytica* [214], constitute useful tools to guide the manipulate of yeast metabolism [215,216]. Another advantage of in silico approaches is the identification of targets that may be relevant for increasing stress tolerance. For example, the entire transportome of *Starmerella bombicola* was unveiled using a bioinformatics tool that identifies putative transporters and the obtained results highlighted the role of the ABC transporters superfamily in the export of sophorolipids [217].

### 7.2. Genetic Engineering to Improve Lipid Biosynthesis

Enzymes involved in the lipid biosynthetic pathway are likely key molecular targets for the development of strategies to improve yeast oil accumulation. One of the most widely used approaches relies on the increase in the expression of genes encoding enzymes that directly influence oil accumulation (Table 6). Two of the most explored molecular targets are the diacylglycerol O-acyl-transferases (*DGA*) encoding genes. The enzyme Dga1 was characterised in different yeast species and its overexpression promotes lipid accumulation [218,219,220,221,222,223]. In *Y. lipolytica*, two different coding genes, *DGA1* and *DGA2*, were identified, but Yl*DGA1* outperforms Yl*DGA2* [224]. Another approach involves the redirection of the central carbon metabolism to increase the availability of precursors by the over-expressing malic enzyme (ME) and acetyl-CoA carboxylase (ACC) that supply the pathway with the essential molecules, acetyl-CoA, NADPH, and malonyl-CoA, respectively (Table 6). Since the malic enzyme of *R. toruloides* is the main enzyme providing NADPH during synthesis, its expression is essential for lipid accumulation [225], whereas its overexpression in *Y. lipolytica* or *L. starkeyi* does not alter lipid accumulation [226,227]. In *Y. lipolytica*, the main source of NADPH is the pentose phosphate pathway [228]. To surpass this limitation, four biosynthetic pathways were designed to convert NADH into NADPH in *Y. lipolytica*. The best result was obtained for a *Y. lipolytica* strain co-overexpressing the glyceraldehyde-3-phosphate dehydrogenase GapC (catalyses the conversion of glyceraldehyde 3-phosphate to 1,3-diphosphoglycerate with the reduction of NAD to NADH) and malate dehydrogenase, the enzyme encoded by the *Mucor circinelloides MCE2* gene; this enzyme is responsible for the decarboxylation of malate with reduction of NADP^+^ to NADPH (Table 6) [229]. The overexpression in *Y. lipolytica* and *R. toruloides* of acetyl-CoA carboxylase, encoded by *ACC1* which catalyses the carboxylation of acetyl-CoA to malonyl-CoA, led to an increased lipid content [221,230]. On the other hand, the homologous or heterologous overexpression of ATP-citrate lyase (*ACL*) genes, responsible for the supply of acetyl-CoA from the cleavage of citrate, did not lead to an increase in lipid content [231]. Other genetic manipulations were also explored, including the manipulation of fatty acid synthesis to obtain lipids with longer chains or targeting the expression of acyl-CoA/acyl-ACP processing enzymes in other cellular compartments, such as the cytoplasm, the peroxisome or the endoplasmic reticulum, in order to minimise the effects of compartmentalisation on the accessibility to the downstream engineered biocatalysts [232].

The deletion of genes involved in lipid degradation, such as the acyl-CoA oxidases (*POX*) or peroxisomal biogenesis (*PEX*) genes, was also examined. The deletion of one of these genes, *PEX10*, to abolish peroxisome biogenesis and therefore lipid catabolism, enhanced lipid accumulation in *Y. lipolytica* [233], while in *R. toruloides* lipid accumulation was reduced [225]. The elimination of *PEX10* also led to slower growth, corroborating previous studies that found that peroxisome biosynthesis is required for robust cell growth in basidiomycetes [234,235,236]. The deletion of genes related to by-product formation was also found to be beneficial to lipid accumulation. For example, this is the case of the elimination of genes of glycogen synthesis, indicating that this pathway competes with oil accumulation [237].

Transcription factor engineering also emerged as a promising strategy to increase yeast tolerance to different biotechnological relevant stresses to be used with the goal of enhancing lipid production. To assure a C/N ratio suitable for lipid production, high concentrations of glucose are usually present and consequently, the expression of genes required for the metabolism of alternative carbohydrates, gluconeogenesis and mitochondrial functions are repressed [238,239]. The deletion of the transcription factor *MIG1* [239] or the mutation of *SNF1*, encoding a serine/threonine-protein kinase that regulates *MIG1* [240], both involved in glucose repression, was found to increase lipid titers in *Y. lipolytica.* Furthermore, the deletion of *MGA2*, a regulator of the expression of desaturases, enhanced lipogenesis and the biosynthesis of unsaturated fatty acids [241]. The importance of fatty acid desaturases as potential targets for increasing lipid accumulation was widely examined. The expression of these membrane-bound proteins that catalyse the addition of a double bond in fatty acid hydrocarbon chains to produce unsaturated and polyunsaturated fatty acids, was found to enhance lipid production in several yeast species/strains as well as the accumulation of unsaturated lipids, which is beneficial for biodiesel production [223,225,242,243,244].

**Table 6:** Genetic modifications performed in different oleaginous yeasts to increase lipid production. A brief description of the experimental conditions and genetic modification, as well as lipid production, is also presented. The species covered in the table are *Yarrowia lipolytica*, *Rhodotorula toruloides*, *Rhodotorula fluvialis* and *Candida phangngensis*. The underlined values correspond to the lipid production parameters obtained for the parental strain.

Species and Strain	Experimental Condition	Genetic Modification	Lipid Content (%) Lipid Titer (g/L) Lipid Productivity (g/Lh)	Ref.
*Y. lipolytica* JMY3501, derived from Po1d	Bioreactor, 150 g/L Suc, 1.7 g/L YNB, 3.75 g/L NH_4_Cl, 0.7 g/L KH_2_PO_4_, and 1.0 g/L MgSO_4_.7H_2_O. C/N 60.	*pox1- 6***Δ** and *tgl4***Δ**. **o/e** of *GDP1* and *DGA2*	- 5.76 -	[245]
*Y. lipolytica* Y4086, derived from Po1d		*pox1- 6***Δ** and *tgl4***Δ**. **o/e** of *GDP1*, *DGA2,* and *HXK1.* **h.e.** of Sc*SUC2*	- 9.15 -	
*Y. lipolytica*, derived from Po1f	Test-tube, 80 g/L Glu, 6.7 g/L YNB^(−/−)^, 1.365 g/L NH_4_, 0.79 g/L CSM supplement.	*pex10***Δ** and *mfe1***Δ**. **o/e** of *DGA1*	74/16.8 6.0/0.41 -	[224]
*Y. lipolytica*, derived from Po1f	Bioreactor (*batch*), 160 g/L Glu, 13.4 g/L YNB^−/−^, 2.73 g/L NH_4_.		70.6/- - -	
*Y. lipolytica* MTYL065, derived from Po1g	Bioreactor, 90 g/L glucose, 1.5 g/L YNB^−/−^, 2 g/L (NH₄)₂SO₄, 1 g/L YE.	**o/e** of *ACC1* and *DGA1*	61.7/11.7 - 0.143/-	[230]
*Y. lipolytica* MTYL065, derived from Po1g	Flask, 50 g/L Glu, 1.7 g/L YNB^−^^/−^, 1.5 g/L YE, C/N 20.	**o/e** of *ACC1* and *DGA1*	41.4/8.77 - -	[230]
*Y. lipolytica* YL-ad9, derived from Po1g	Bioreactor, 150 g/L Glu, 3.4 g/L YNB^−/−^, 8.8 g/L (NH₄)₂SO₄, 2 g/L YE.	Replacement of the hp4d promoter with the TEFin promoter to co-overexpress *ACC1* and *DGA1* **o/e** of stearoyl-CoA desaturase (*SCD*)	67/- 55/- 0.707/-	[223]
*Y. lipolytica* Yl*gsy1***Δ**, derived from H222	Bioreactor, 20 g/L Glu, 0.4 g/L (NH₄)₂SO₄.	*gsy1* **Δ**	>60% TAGs synthesis increase	[237]
	Bioreactor, 20 g/L Gly, 0.4 g/L (NH₄)₂SO₄.			
*Y. lipolytica snf1****Δ***, derived from ATCC 20362	Flask, Growth phase: SD medium (2% Glu and 0.5% (NH₄)₂SO₄).	*snf1***Δ** Expression of **Δ**- 9 elongase/**Δ**-8 desaturase pathway	18.5/7.1 - -	[240]
*Y. lipolytica snf1****Δ***, derived from ATCC 20362	Flask, Oleaginous phase: high concentration of Glu (8% Glu without N source).		18.5/12.6 - -	
*Y. lipolytica* M25, derived from ACA-DC 50109	Flask, 5.0% Glu, 0.7% KH_2_PO_4_, 0.25% Na_2_HPO_4_, 0.15% MgSO_4_·7H_2_O, 0.015% CaCl_2_, 0.015% FeCl_3_·6H_2_O, 0.002% ZnSO_4_·7H_2_O, 0.006% MnSO_4_·H_2_O, 0.05% YE.	*mig1* **Δ**	48.7/36.0 - -	[239]
*Y. lipolytica* L36 *DGA1*, derived from PO1f	Bioreactor *(fed-batch*), 80 g/L Glu, 3.4 g/L YNB^−/−^, 4 g/L (NH₄)₂SO₄.	Mutation in the gene *MGA2* (mga2-G643R). **o/e** of *DGA1*	- 25/- 0.145/-	[241]
*Y. lipolytica* NS432, derived from YB-392	Bioreactor (*batch*), 150 g/L Glu, 0.1 g/L corn peptone, 3 g/L YE.	**o/e** of *DGA1* from *R. toruloides* **h.e.** of *DGA2* from *Claviceps purpurea* *tgl3***Δ**	77/24 30.8/3.6 0.27/0.03	[222]
*Y. lipolytica* NS432, derived from YB-392	Bioreactor (*fed-batch*), 150 g/L Glu, 0.1 g/L corn peptone, 3 g/L YE.		73/25 84.5/12.8 0.73/0.11	
*Y. lipolytica* YL10, derived from PO1f	Bioreactor, 40 g/L Glu, 1.7 g/L YNB^−/−^, 3.52 g/L (NH₄)₂SO₄, 2 g/L uracil.	**h.e.** of ***Δ****-15* desaturase from flax **o/e** of *ACC1*, *DGA1*, *SCD*, **Δ**-12D *pex10***Δ** and *mfe1***Δ**	77.8/- 50.0/- -	[244]
*Y. lipolytica* VHb, derived from Polh	Bioreactor, 90 g/L Glu, 1.5 g/L YNB^−/−^, 2 g/L (NH₄)₂SO₄, 1 g/L YE.	**h.e.** of *Vitreoscilla* haemoglobin (VHb)	14.5/10.5 - -	[246]
*Y. lipolytica* YL-1292sp-ACL-6, derived from Polh	Flask., Modified K&R medium with 100 g/L Gly, 2 g/L C_4_H_12_N_2_O_6__._	**h.e.** of ACL from *Mus musculus*	23.1/7.3 - -	[247]
*Y. lipolytica* AD-per*CAT2,* derived from Polg	Bioreactor, 100 g/L Glu, 3.4 g/L YNB^−/−^, 2.2 g/L (NH₄)₂SO₄.	**h.e.** of per*CAT2* from *Saccharomyces cerevisiae* **o/e** *ACC1* and *DGA1*	- 66.4/- 0.565/-	[232]
*Y. lipolytica* Adgapc, derived from Polg	Bioreactor, 100 g/L Glu, 3.4 g/L YNB, 2.5 g/L YE, 8.8 g/L (NH₄)₂SO₄	**h.e.** of GapC from *Clostridium acetobutylicum*	62.5/54.7 63.3/47.8 -	[229]
*Y. lipolytica* Adme, derived from Polg		**h.e.** of *MCE2* from *Mucor circinelloides*	63.7/54.7 61.4/47.8 -	
*Y. lipolytica* Adpp, derived from Polg		Co-expression of a phosphoketolase from *Leuconostoc mesenteroides* and a phosphate acetyltransferase from *Clostridium kluyveri*	52.7/54.7 56.2/47.8 -	
*Y. lipolytica* Adgy, derived from Polg		Co-expression of the heterologou *Clostridium acetobutylicum* GapC and *Y. lipolytica YEF*	63.2/54.7 54.6/47.8 -	
*Y. lipolytica* Adgm, derived from Polg		Co-expression of the heterologous *Clostridium acetobutylicum* GapC and the heterologous *Mucor circinelloides MCE2*	75.5/54.7 66.8/47.8 -	
*Y. lipolytica* ALDH, derived from Polg	Bioreactor, 100 g/L Glu, 3.4 g/L YNB^−/−^, 4.4 g/L (NH₄)₂SO₄.	**o/e** of glutathione peroxidase (*GPO*), glutathione disulfide reductase (GSR), **h.e.** of glucose-6- phosphate dehydrogenase (*S.cerevisiae* Zwf1) and aldehyde dehydrogenase (*Escherichia coli* AldH)	81.4/40.6 72.7/- 0.97/-	[248]
*Y. lipolytica* JMY5035, derived from Pold	Flask., 6% Soluble starch, 0.17% (*w*/*v*) YNB, 0.15% (*w*/*v*) NH_4_Cl, pH 6, C/N 60.	**o/e** of *DGA2, GPD1* and **h.e.** of alpha-amylase from *Oryza sativa* + glucoamylase from *Aspergillus niger*; *pox1-6***Δ** and *tgl4***Δ**	21.1/3.7 2.44/- -	[249]
	Flask, 6% Soluble starch, 0.17% (*w*/*v*) YNB, 0.15% (*w*/*v*) NH_4_Cl, pH 6, C/N 90.	**o/e** of *DGA2, GPD1* and **h.e.** of alpha-amylase from *Oryza sativa* + glucoamylase from *Aspergillus niger*; *pox1-6***Δ** and *tgl4***Δ**	27.0/- 3.32/- -	
*R. toruloides* RT880-AD, derived from IFO 0880	Bioreactor (*batch*), 150 g/L Glu, 0.5 g/L (NH_4_)_2_SO_4_, 1 g/L KH_2_PO_4_, 1 g/L MgSO_4_, 8 g/LYE.	**o/e** of *ACC1* and *DGA1*	53.9/36.0 24.8/14.2 0.25/0.09	[225]
*R. toruloides* RT880-ADS, derived fromRT880-AD		**o/e** of *ACC1*, *DGA1*, and SCD	51.1/36.0 27.4/14.2 0.31/0.09	
*R. toruloides* RT880-ADM, derived fromRT880-AD	Flask, N-limited medium supplemented with 70 g/L Glu.	**o/e** of *ACC1*, *DGA1* and malic enzyme	- 18.6/16.5 -	
*R. toruloides* TK16	Flask, 70 g/L Glu, 0.55 g/L (NH_4_)_2_SO_4_, 0.4 g/L KH_2_PO_4_, 2 g/L MgSO_4_.7H_2_O, 0.75 g/L YE	**o/e** of **Δ***12-FAD*	27.0/16.0 5.9/2.5 -	[243]
*R. toruloides* TK16	Flask, 70 g/L Glu, 0.55 g/L (NH_4_)_2_SO_4_, 0.4 g/L KH_2_PO_4_, 2 g/L MgSO_4_.7H_2_O, 0.75 g/L YE	**o/e** of **Δ***9-FAD +* **Δ***12-FAD*	26.0/16.0 3.5/2.5 -	[243]
*R. toruloides* L1-1		**o/e** of **Δ***12-FAD*	24.0/14.8 6.7/4.5 -	
*R. toruloides* L1-1		**o/e** of **Δ***9-FAD +* **Δ***12-FAD*	20.0/14.8 6.0/4.5 -	
*R. toruloides* NP-Pta-15, derived from NP11	Flask, 50 g/L Glu, 1.5 g/L Mg_2_SO_4_.7H_2_O, 0.1 g/L (NH_4_)_2_SO_4_, 0.75 g/L YE.	**o/e** of phosphotransacetylase (Pta)	65.6/62.1 - 0.05/0.03	[250]
*R. fluvialis* DMKU-RK253	Flask, 70 g/L crude Gly, 0.55 g/L (NH_4_)_2_SO_4_, 1 g/L MSG, 2 g/L MgSO_4_.7H_2_O, 0.4 g KH_2_PO_4._	**o/e** of *DGA1*	18.53/6.11 1.2/0.47 -	[220]
*C. phangngensis* JQCP03H, derived from PT1-17	Flask, Lipid production medium. 50 g/L Glu, 4 g/L pep, 1.5 g/L YE.	**h.e.** of *DGA1* from *Y. lipolytica*	63.3/52.1 11.4/8.3 -	[219]

Notes: -, no data available. **h.e.**: heterologous expression; **o/e**: overexpression; **Δ**: deletion. Lipid content: g of produced lipids/g dry weight (%). Lipid titer: g of produced lipids/L of culture. Lipid productivity: g of produced/L of culture per hour. Abbreviations: Glycerol (Gly); Glucose (Glu); Monosodium glutamate (MSG); Sucrose (Suc); References (Ref.); YE: yeast extract; YNB-/-: yeast nitrogen base without amino acids and without ammonium sulfate.

### 7.3. Genetic Engineering of Substrate Utilisation Pathways, in Particular of Xylose

Numerous genetic manipulations were performed to increase the efficiency of consumption of substrates of difficult catabolism. As referred to in Section 4.2, *Y. lipolytica* possesses in its genome genes encoding xylose reductase (XR), xylitol dehydrogenase (XDH) and xylulose kinase (XK), but they are not sufficiently expressed to allow the efficient catabolism of xylose [125]. To address this issue, the heterologous expression of xylose pathway genes from microorganisms that use this sugar as a C-source, such as the yeast *Scheffersomyces stipitis*, was attempted (Table 7). Interestingly, the most efficient xylose consumption was obtained using a lipid-accumulating strain, referred to as the obese strain (overexpresses the G3P dehydrogenase *GPD1* and the diacylglycerol O-acyl-transferase *DGA2* genes and has the genes that code for the acyl-CoA oxidases (*POX1-6*) and the triacylglycerol lipase, *TGL4*, deleted) overexpressing the genes encoding the xylose reductase and xylitol dehydrogenase from *S. stipitis* and the xylulose kinase from *Y. lipolytica* [54].

The expression of genes involved in xylose metabolism, namely the endogenous phosphoketolase (PK) and the expression of a heterologous phosphotransacetylase (PTA) in *Rhodosporidium azoricum* also led to an increase of 89% in lipid yield using a culture medium with a mixture of glucose and xylose, without compromising biomass production and improving xylose utilisation [251]. As discussed in Section 4.2, xylose transport constitutes a limiting step in xylose utilisation. Transporters that allow the co-consumption of glucose and xylose were identified in *C. tropicalis* [29] and *L. starkeyi* [110] and may be considered targets for future genetic engineering of oleaginous yeasts. A new family of transporters very abundant in plant genomes is of potential interest. They belong to the Sugars Will Eventually be Exported Transporter (SWEET) superfamily and present a wide-ranging specificity and affinity towards a variety of mono- and disaccharide sugars [110,252]. Due to the high affinity for glucose and xylose of the transporters of this superfamily, they are pointed out as promising regarding the co-utilisation of both sugars being considered good targets for genetic manipulation of *S. cerevisiae* and other biotechnologically relevant yeasts [252].

Another sugar of more difficult catabolism is the acid sugar D-galacturonic acid, a monomer of pectin abundant in residues rich in pectin. Species of the *Rhodotorula* genus are able to efficiently catabolise D-galacturonic acid [39] but this is not the case for most of the yeast species, in particular *S. cerevisiae* [40]. A recent study involved the engineering of D-galacturonic acid catabolism in an *S. cerevisiae* strain previously equipped with a NAD-dependent glycerol catabolic pathway [253]. Although this study has proved the capacity to produce bioethanol, not lipids, from D-galacturonic acid in *S. cerevisiae*, results can be considered a proof of concept for the use as feedstocks two industrial organic residues/by-products such as the pectin-rich residues sugar beet pulp from sugar refinery or citrus peels, and crude glycerol, from the biodiesel industry [253].

**Table 7:** Genetic modifications performed in *Yarrowia lipolytica* to improve xylose consumption. Strains of *Y. lipolytica* that are not genetically manipulated and not able to use xylose as C-source are considered controls. The meaning of the abbreviations used is explained at the end of the table. The underlined values correspond to the lipid production parameters obtained for the parental strain.

Strain	Experimental Condition	Genetic Modification	Consumed Xylose (g/Lh)	Lipid Content (%) Lipid Titer (g/L) Lipid Productivity (g/Lh)	Ref.
**XYL+**, derived from Po1d	Bioreactor (*fed-batch*), 150 g/L Xyl, 1.6 g/L NH_4_Cl, 1 g/L YE, 1 g/L YNB, 1.0 g/L MgCl_2_.7H_2_O, 0.5 g/L H_2_PO_4._	**o/e** of *XK* **h.e.** of *XR* and *XDH* from *Scheffersomyces stipitis*	2.14	- 5.9 0.06	[54]
**XYL+Obese**, derived from Po1d		**o/e** *XK*, *DGA2* and *GPD1* **h.e.** of *XDH* and *XR* from *Scheffersomyces stipitis pox1-6***Δ** and *tgl4***Δ**		- 20.1 0.19	
**XYL+**, derived from Po1d	Bioreactor (*fed-batch*), co-feeding with Gly, 150 g/L Xyl, 1.6 g/L NH_4_Cl, 1 g/L YE, 1 g/L YNB, 1.0 g/L MgCl_2_.7H_2_O, 0.5 g/L H_2_PO_4._	**o/e** of *XK* **h.e.** of *XR* and *XDH* from *Scheffersomyces stipitis*	-	- 7.3 0.03	
**XYL+Obese**, derived from Po1d	Bioreactor (*fed-batch*), co-feeding with Gly, 150 g/L Xyl, 1.6 g/L NH_4_Cl, 1 g/L YE, 1 g/L YNB, 1.0 g/L MgCl_2_.7H_2_O, 0.5 g/L H_2_PO_4._	**o/e** of *DGA2*, *GDP1*, *XK* **h.e.** of *XR* and *XDH* from *Scheffersomyces stipitis* *pox1-6***Δ** and *tgl4***Δ**	-	- 50.5 0.23	
**YlXYL+Obese-XA**, derived from Po1d	Bioreactor (*fed-batch*), Lignocellulosic hydrolysate from agave with 18% Glu and 12% Xyl, C/N 15.	**o/e** of *XDH*, *XR*, *XK, DGA2* and *GPD1* **h.e.** *XPKA* and *ACK* from *Aspergillus nidulans* *pox1-6***Δ** and *tgl4***Δ**	0.47	67 16.5/2.0 0.185	[254]
**E26 XUS**, derived from E26	Bioreactor (*batch*),160 g/L Xyl, YNB with 10 g/L (NH₄)₂SO₄.	**h.e.** of *XYL1* and *XYL2* from *Scheffersomyces stipitis*	0.74	- 15.1 0.19	[255]
**YSXID**, derived from Po1f	Bioreactor (*batch*), 80 g/L Glu and 80 g/L Xyl, 0.69 g/L CSM Leu/Ura, 1.76 g/L YNB^−/−^, 3.52 g/L (NH₄)₂SO₄, C/N 100.	Genetic background YSX (obtained in an ALE experiment) **o/e** of *DGA1* and XK **h.e.** of xylose isomerase mutant gene *pex10***Δ**	0.08	56.7/51.6 13.5/7.3 -	[256]
**YSXID**, derived from Polf	Bioreactor (*fed-batch*), Lignocellulosic hydrolysate from *Miscanthus sacchariflorus* supplemented with Glu and Xyl at a final concentration of 35 g/L.	YSX background (obtained in an ALE experiment) **o/e** of *DGA1* and *XK* **h.e.** of xylose isomerase mutant gene *pex10***Δ**	-	42.4/- 12.01/- -	[256]
**PSA02004PP**, derived from PSA02004	Bioreactor (*batch*), Minimal medium with 60g/L Xyl.	**o/e** of *XR*, *XDH* and *XK*.	0.71	- - -	[257]
**Y14**, derived from ATCC 201249	Bioreactor (*batch*), YPX (2% Xyl).	*ku70***Δo/e**. of *XKS,* tHMG1, *ERG9*, *ERG20*, TKL, TAL^1^, TX. **h.e.** of *DS*, *PPDS, ATR1* **h.e.** of *XYL1* and *XYL2* from *Scheffersomyces stipitis* Adaptation step in xylose.	0.56	- - -	[258]
**YBX08**, derived from PDe1	Flas, YP with 40 g/L Xyl.	**o/e** of *XK*, *tLS, tNDPS*. *HMG1* and *ERG12* **h.e.** of *XR* and *XDH* from *Scheffersomyces stipitis*	~0.56	- - -	[259]
**Yl -nar05**, derived from Po1f	Flask, YPX medium, with 40 g/L Xyl.	**o/e** of *XDH*, *XKS* and TAL^2^.	0.56	- - -	[260]

Notes: -, no data available. **h.e.**: heterologous expression; **o/e**: overexpression; **Δ**: deletion. Lipid content: g lipids/g dry weight (%); Lipid concentration: g of produced lipids/L of culture; Lipid yield: g of produced lipids/L of culture per hour. Abbreviations: Acetate kinase (ACK); DS (DMD synthase); *DGA2* (acyl-CoA: diacylglycerol acyltransferase); *GPD1* (G3P dehydrogenase); *ERG12* (mevalonate kinase); *HMG1* (HMG-CoA reductase gene); Phosphoketolase (XPKA); *POX1-6* genes (acyl-CoA oxidases); PPDS (PPD synthase) tLS (d-limonene synthase from *Agastache rugosa*); TAL^1^ (transaldolase); TAL^2^ (tyrosine ammonia lyase); TKL (transketolase); tNDPS (neryl di-phosphate synthase 1 from *Solanum lycopersicum*); TX (xylose transporter); *TGL4* (Triacylglycerol lipase 4); Xylitol dehydrogenase (XDH); Xylulose kinase (XK), Xylose reductase (XR) and YNB^−/−^ (yeast nitrogen base without amino acids and without ammonium sulphate).

### 7.4. Genetic Engineering to Increase Yeast Tolerance to Stress Factors

The unveiling of the molecular mechanisms and functional pathways involved in yeast cell response to toxicants is essential to guide the genetic manipulation of oleaginous yeasts to improve tolerance. The use of lignocellulosic and industrial organic residues biomass for the production of added-value chemicals is a challenging task since yeast cells need to cope with multiple bioprocess-related stresses, either individually or combined, emphasising the relevance of enhancing multiple stress tolerance to maximise their performance in industrial production [89,261,262,263,264]. Physical and chemical extracellular stresses include non-optimum ranges of temperature and pH, osmotic pressure and the presence of growth inhibitors [89,261,262,263,264]. Despite being considered synonyms in some contexts, the concepts of tolerance and robustness may not coincide. Tolerance is defined as the ability of a cell to grow in the presence of single or multiple perturbations whereas the robustness concept is based on the stability of specific phenotypic traits in a multi-stress environment [136]. Thus, having tolerant and robust strains is fundamental for developing the sustainable production of lipid-based biofuels by yeasts. Moreover, yeast resilience, i.e., yeast’s ability to recover from a large environmental perturbation [265], is also important in the biorefinery context.

Concerning oleaginous yeasts, the individual and combined effect of six inhibitors from three major groups of inhibitors (furaldehydes, aromatics and weak acids) was investigated in *Y. lipolytica* overexpressing the endogenous xylose reductase, xylitol dehydrogenase, and xylulose kinase grown in glucose and in xylose [94]. The obtained results were similar in both C-sources, being cinnamic acid and coniferyl aldehyde tolerated while furfural contributed to an extended lag phase and hydroxymethylfurfural was responsible for partial growth inhibition [94]. Formic acid only compromised growth at concentrations above 25 mM [94]. A recent study identified that the native furfural detoxification mechanism and furfural resistance were increased through the rational engineering of *Y. lipolytica*, by the overexpression of aldehyde dehydrogenase endogenous genes to enhance the conversion of furfural to furoic acid [266]. The most promising result was obtained for the aldehyde dehydrogenase *FALDH2*, leading to the highest conversion rate of furfural to furoic acid, as well as a two-fold increase in cell growth and lipid production in the presence of 0.4 g/L of furfural [266]. The thermotolerant L1–1 strain of *R. toruloides*, obtained by an adaptive breeding strategy [267], was also found to tolerate (i) oxidative stress (ethanol and hydrogen peroxide), (ii) osmotic stress (high glucose concentrations), and (iii) cell membrane disturbing reagent (DMSO) [268]. This strain, which produced high titers of lipids, was able to cope with the increase in ROS and presented a stronger cell wall and increased levels of unsaturated membrane lipids under various stresses [268].

It is known that growth inhibitors present in lignocellulosic hydrolysates may compromise the integrity, fluidity and selective permeability of yeast plasma membrane [269]. For this reason, the majority of membrane engineering attempts to increase tolerance to multiple stresses target the modulation of its lipid composition, in order to maintain the integrity and fluidity under stress, namely by altering lipid saturation or changing the length of lipid in biomembranes [264]. The genetic manipulation of oleaginous yeasts, comprising the degree of saturation of lipids [200,223,243,244,270,271,272] or the length of the lipidic chain [273,274] led to increased lipid titers. Additionally, membrane proteins including integral membrane proteins and transport proteins are also extremely relevant in stress tolerance. A remarkable example is overexpression in *Y. lipolytica* of the gene *MFS1*, a putative MFS transporter, that led to an increased propionate tolerance [275]. However, studies on the role of transporters in stress tolerance are scarce in oleaginous yeasts but this is a research topic explored in the yeast model *S. cerevisiae*. A relevant example regarding transport proteins and stress level involves *TRK1*, encoding the high-affinity potassium transporter and a major determinant of tolerance to acetic acid in *S. cerevisiae* [84]. Potassium supplementation up to the required level was described as involved in the tolerance to a wide variety of stresses [84,276] and, recently, it was demonstrated that *S. cerevisiae* cells react to potassium concentration by a rapid, continuous, and precise adjustment of both the affinity and maximum velocity of their Trk1 protein [277]. However, the role of Trk1 in tolerance to different stresses is not completely clear: under formic acid (C1) stress, the deletion of *TRK1* led to increased tolerance to formic acid, contrasting with what was described for acetic acid and the demonstrated role that K+ concentration has in tolerance [278]. The relevance of plasma membrane efflux pumps in the development of superior yeasts was recently reviewed [135]. Furthermore, the genetic alterations (gene deletion or overexpression) with a direct effect on the tolerance of *S. cerevisiae* towards individual inhibitors or combinations of inhibitors found in lignocellulosic hydrolysates or other feedstocks of interest in the context of a circular bio-economy were compiled in several works [81,89,262,279,280,281]. For example, the overexpression of Ace2, a transcription factor required for septum destruction after cytokinesis and Sfp1, a transcription factor that regulates ribosomal protein and biogenesis genes in *S. cerevisiae*, was beneficial to increase tolerance to acetic acid, furfural, and a mixture of acetic acid and furfural [282]. Regarding another example of transcription factor engineering, a single amino acid exchange at position 135 (serine to phenylalanine) in Haa1, a major TF involved in adaptation and tolerance to acetic and formic acids stresses [85,283], contributed to an increase in acetic acid tolerance [284].

### 7.5. Adaptive Laboratory Evolution (ALE) to Improve Yeast Robustness and Substrate Utilisation

Adaptive laboratory evolution (ALE) techniques [285,286] are a suitable alternative to the use of genetic engineering when the necessary efficient tools are not available as is the case for most of the non-*Saccharomyces* yeasts and they also constitute a highly convenient strategy to complement genetic manipulation experiments in *Y. lipolytica* [256,287,288]. The major objectives for such adaptation are to increase in tolerance of the producing yeasts to the inhibitors present in lignocellulosic hydrolysates or other residual feedstocks and the efficiency of substrate utilisation [285,286]. Of course, in the specific context of this review paper, this is an essential objective to assure yeast robustness [136] under the above-referred challenging conditions or, in other words, to maintain the high levels of oil production in the evolved strains. For example, a genetically manipulated strain of *R. toruloides* was adapted to undetoxified wheat straw hydrolysates leading to a higher rate of xylose consumption [289]. The genes *DGAT1* (diacylglycerol acyl-CoA transferase type 2) and *SCD1* (stearoyl-CoA desaturase), under the control of the xylose reductase (*XYL1*) promoter, previously reported to enhance lipid production in oleaginous yeasts [230,289] were overexpressed in this improved strain, leading to a lipid concentration of 39.5 g/L and lipid productivity of 0.334 g/Lh, representing the highest values described in the literature [289]. Considering the tolerance to the inhibitors present in lignocellulosic hydrolysate, an ALE attempt also using *R. toruloides* was performed to increase tolerance to HMF, furfural, acetic acid, and better performance for the accumulation of lipids and carotenoids was obtained [290]. Increased tolerance to inhibitors present in lignocellulosic biomass, either alone (in the case of formic acid) or in a mixture of inhibitors (formic acid, acetic acid, furfural and HMF) in *Metshnikowia pulcherrima* was also obtained using ALE, leading to a decreased duration of the latency period and an increased specific growth rate after growth resumption [291]. The lipid content was also increased by 50% in the evolved strain compared to the parental strain [291].

## 8. Conclusions and Future Perspectives

The efficient use of yeasts as cell factories for the production of lipids from lignocellulosic biomasses or other residual feedstocks is a major challenge in the transition towards a sustainable and low-carbon bio-economy. The genetic and physiological diversity of oleaginous yeasts is an advantage for the transition to greener biofuels given that some of them are able to efficiently consume all the carbon sources present in those feedstocks and tolerate the growth and metabolism inhibitors that are present or that result from their pre-treatment. In order to increase the performance of lipid production, it is crucial to match the oleaginous yeast species/strain to be used in the chosen feedstock. Although *Rhodotorula toruloides* and *Yarrowia lipolytica* stand out in the scientific literature as the most studied and promising yeast species, strain performance is highly variable and other yeast species/strains are also emerging as highly promising. However, while *R. toruloides* is characterised to efficiently utilise most of the sugars and other carbon sources present in biomass hydrolysates, has a high lipid production yield and is robust, *Y. lipolytica* allows easy genetic manipulation since several efficient genetic tools are already available. Multidisciplinary approaches, combining and integrating data from genome-wide analyses, the exploration of metabolic models and a holistic understanding of the physiology of these yeasts are expected to guide the rational construction of yeasts with superior characteristics and their use under appropriate conditions. This will contribute to rendering current biodiesel production greener and making the bioprocess more economically sustainable.

It is worth mentioning that the fatty acids from the TAGs, produced by native and engineered oleaginous yeast strains by modifying the lipid profile toward other chain lengths and saturation types, can also be used to produce other products than biodiesel for higher value markets (pharmaceuticals, nutraceuticals, cosmetics, food) [231,292,293,294].

## Figures and Tables

**Table 1:** Lipid production by various oleaginous yeasts based on hydrolysates of lignocellulosic biomasses. The species contemplated in this analysis were: *Cryptococcus aerius*, *Cryptococcus albidus*, *Cryptococcus curvatus*, *Cryptococcus humicola*, *Lipomyces kononenkoae*, *Lipomyces starkeyi*, *Lipomyces tetrasporus*, *Rhodotorula glutinis*, *Rhodotorula graminis*, *Rhodotorula. paludigenum*, *Rhodotorula. taiwanensis*, *Rhodotorula toruloides*, *Rhodosporidiobolus fluvialis*, *Saitoella* *coloradoensis*, *Saitoella* *complicata*, *Trichosporon cutaneum*, *Trichosporon dermatis*, *Trichosporon guehoae*, *Trichosporon oleaginosus*, and *Yarrowia lipolytica*.

Species and Strain	Feedstock and Bioprocess Type	Lipid Content (%) Lipid Titer (g/L) Lipid Productivity (g/Lh)	Ref.
*C. aerius* Y-1399	Corn stover hydrolysate. Using 96-well plate, 25 °C, 400 rpm, pH 6.0, C/N 62:1, 59.3 g/L Glu, 36.3 g/L Xyl, 5.6 g/L Ara, 0.6 mM HMF, 0.09 mM Fur, 2.4 g/L AcA.	- 9.8 0.089	[60]
*C. albidus* ATCC 10672	Sorghum stalk hydrolysate. Flask, 25 °C, 200 rpm, 51% Glu, 30% Xyl, 2.9% Ara.	42.0 4.6 -	[61]
	Switchgrass hydrolysate. Flask, 25 °C, 200 rpm, 58% Glu, 26% Xyl.	44.0 4.7 -	
*C. curvatus* ATCC 20509	Corn stover hydrolysate. Bioreactor, 30 °C, pH 5.2, 1 vvm, 25% DO, 97.47 g/L Glu, 58.02 g/L Xyl, 3.51 g/L Gal, 8.56 g/L Ara, 0.24 g/L Fruc. Supplementation with 2.00 g/L YE, 4.00 g/L pep and 1.60 g/L YNB.	63.1 21.4 0.220	[62]
	Wheat straw hydrolysate. Flask, 28 °C, 200 rpm, 3.2 g/L Glu, 14.0 g/L Xyl, 3.7 g/L Ara, 0.8 g/L Gal, 4.2 g/L AcA, 0.03 g/L Fur and 0.02 g/L HMF.	27.1 4.2 -	[36]
*C. humicola* UCDFST 10-1004	Corn stover hydrolysate. Flask, 30 °C, 200 rpm, pH 5.5, 63.2 g/L Glu, 28.9 g/L Xyl.	40.0 15.5 -	[63]
*L. kononenkoae* Y-7042	Corn stover hydrolysate. Using 96-well plate, 25 °C, 400 rpm, pH 6.0, C/N 62:1, 59.3 g/L Glu, 36.3 g/L Xyl, 5.6 g/L Ara, 0.6 mM HMF, 0.09 mM Fur, 2.4 g/L AcA.	- 11.3 0.081	[60]
*L. starkeyi* DSM 70296	Sugarcane bagasse hydrolysate. Flask, 28 °C, 200rpm, pH 5.5, C/N 50, 13.1 g/L Xyl, 2.2 g/L Glu, 2.1 g/L AcA, 2.2 g/L Ara, 0.02 g/L Fur, 0.02 g/L HMF.	26.9 - -	[64]
	Sugarcane bagasse hydrolysate. Bioreactor, 28 °C, 400rpm, pH 5.5, 1 vvm, C/N 50, 13.1 g/L Xyl, 2.2 g/L Glu, 2.1 g/L AcA, 2.2 g/L Ara, 0.02 g/L Fur, 0.02 g/L HMF.	26.1 - -	
	Sugarcane bagasse hydrolysate. Flask, 28 °C, 200 rpm, pH 5.5 <3 g/L Glu, 18.5 g/L Xyl, <3 g/L Ara, 3.6 g/L AcA, Furfural < 300 ppm, HMF < 200 ppm.	27.8 3.5 0.040	[65]
*L. starkeyi* ATCC 56304	Sorghum stalk hydrolysate. Flask, 25 °C, 200 rpm. 51% Glu, 30% Xyl, 2.9% Ara.	44.0 7.9 -	[61]
*L. starkeyi* ATCC 56304	Switchgrass hydrolysate. Flask, 25 °C, 200 rpm, 58% Glu, 26% Xyl.	39.0 6.5 -	[61]
*L. starkeyi* -	Rice straw hydrolysate. Flask, 30 °C, 160 rpm.	36.0 4.6 -	[66]
*L. starkeyi* ATCC 12659	Wheat straw hydrolysate. Flask, 28 °C, 200 rpm, 3.2 g/L Glu, 14.0 g/L Xyl, 3.7 g/L Ara, 0.8 g/L Gal, 4.2 g/L AcA, 0.03 g/L Fur, 0.02 g/L HMF.	29.1 3.7 -	[36]
*L. tetrasporus* Y-11562	Corn stover hydrolysate. Using 96-well plate, 25 °C, pH 6.0, 400 rpm, C/N 62:1. 59.3 g/L Glu, 36.3 g/L Xyl, 5.6 g/L Ara, 0.6 mM HMF, 0.09 mM Fur, 2.4 g/L AcA.	- 11.9 0.100	[60]
*R. glutinis* CGMCC 2.703	Corn stover hydrolysate. Bioreactor, 30 °C, 2.27 g/L Glu, 40.1 g/L Xyl, 0.171 g/L Fur, 0.483 g/L HMF.	36.4 5.5 -	[67]
*R. glutinis* ATCC 204091	Wheat straw hydrolysate. Flask, 28 °C, 200 rpm, 3.2 g/L Glu, 14.0 g/L Xyl, 3.7 g/L Ara, 0.8 g/L Gal, 4.2 g/L AcA, 0.03 g/L Fur and 0.02 g/L HMF.	20.7 2.4 -	[36]
*R. graminis* DBVPG 4620	Corn stover hydrolysate. Bioreactor, 30 °C, 900 rpm, pH 6.0. 126 g/L Glu, 6.5 g/L Gal, 87.1 g/L Xyl, 7.5 g/L Ara, 2.9 g/L Man, 4.9 g/L AcA, 0.46 g/L Fur, 1.85 g/L HMF. Supplementation with CSS, YE and salts.	34.0 - 0.210	[68]
*R. paludigenum* KM281510	Corncob hydrolysate. Flask, 25 °C, 200 rpm, pH 6.0, 54.98 g/L Glu, 19.32 g/L Xyl, 1.13 g/L Ara.	58.4 3.3 -	[58]
*Rhodotorula* sp. KM281508		47.4 2.3 -	
*R. paludigenum* KM281510	Corncob hydrolysate. Bioreactor, 25 °C, 200 rpm, pH 6.5, 54.98 g/L Glu, 19.32 g/L Xyl, 1.13 g/L Ara.	73.0 20.3 0.101	
*R. taiwanensis* AM2353	Corncob hydrolysate. Bioreactor, 26 °C, 200 rpm, pH 7.0, 1.75 L/min aeration, 7.22 g/L Glu, 36.79 g/L Xyl, 0.02 HMF. Supplementation with 0.5% YE.	60.3 - -	[69]
*R. toruloides* ATCC 10788	Wheat straw hydrolysate. Flask, 28 °C, 200 rpm, 3.2 g/L Glu, 14.0 g/L Xyl, 3.7 g/L Ara, 0.8 g/L Gal, 4.2 g/L AcA, 0.03 g/L Fur and 0.02 g/L HMF.	24.6 2.4 -	[36]
*R. toruloides* CCT 7815	Hemicellulosic hydrolysate from Birch. Bioreactor, 30 °C, 400–800 rpm, pH 6.0, DO > 25% 87.1 g/L Glu, 29.8 g/L Gal, 21.0 g/L Man, 298.1 g/L Xyl, 14.1 g/L Ara, 20.5 g/L AcA, 33 g/L phenols	41.0 11.0 -	[70]
*R. toruloides* DSMZ 4444	Corn stover hydrolysate. Bioreactor, 30 °C, pH 5.2, 1 vvm, 25% DO, 97.47 g/L Glu, 58.02 g/L Xyl, 3.51 g/L Gal, 8.56 g/L Ara, 0.24 g/L Fruc. Supplementation with 2 g/L YE, 4 g/L Pep and 1.6 g/L YNB.	60.8 23.3 0.170	[62]
*R. toruloides* Y-1091	Corn stover hydrolysate. Using 96-well plate, 25 °C, 400 rpm, pH 6.0, C/N 62:1, 59.3 g/L Glu, 36.3 g/L Xyl, 5.6 g/L Ara, 0.6 mM HMF, 0.09 mM Fur, 2.4 g/L AcA.	- 8.8 0.095	[60]
*R. toruloides* Y4	Jerusalem artichoke extracts and hydrolysate. Bioreactor, 30 °C, 200–600 rpm, pH 6.0, 40–50% DO.	56.5 39.6 -	[59]
*R. fluvialis* DMKU-SP314	Sugar cane top hydrolysate. Flask, 28 °C, 150 rpm, pH 5.5, 21.4 g/L Glu, 7.1 g/L Xyl, 7.1 g/L unidentified sugars.	43.7 3.4 0.016	[71]
*S. coloradoensis* YB-2330	Corn stover hydrolysate. Using 96-well plate, 25 °C, 400 rpm, pH 6.0, C/N 62:1, 59.3 g/L Glu, 36.3 g/L Xyl, 5.6 g/L Ara, 0.6 mM HMF, 0.09 mM Fur, 2.4 g/L AcA.	- 8.5 0.071	[60]
*S. complicata* Y-17804		- 7.4 0.067	
*T. cutaneum* ATCC 20271	Corncob hydrolysate. Flask, 30 °C, 180 rpm, pH 5.0, 98.9 g/L Glu, 16.6 g/L Xyl.	32.1 12.3 -	[72]
*T. cutaneum* CH002	Corncob hydrolysate. Flask, 28 °C, 150 rpm, pH 7.0, 45.7 g/L sugars, 0.06 g/L Fur, 0.32 g/L HMF, 0.04 g/L AcA, 0.03 g/L ButA.	36.0 7.9 -	[73]
*T. cutaneum* AS 2.571	Corn stover hydrolysate. Flask, 30 °C, 200 rpm, pH 6.0, 60 g/L total sugars.	39.2 7.6 0.078	[74]
*T. dermatis* 32903	Corn stover hydrolysate. Flask, 30 °C, 250 rpm, C/N 110, 43.41 g/L Glu, 22.69 g/L Xyl, 3.79 g/L Ara, 1.82 g/L Cel, 2.32 g/L AcA, 1.32 g/L Fur, 2.62 g/L HMF.	24.2 7.5 0.104	[75]
*T. dermatis* CH007	Corn stover hydrolysate. Flask, 28 °C, 150 rpm, pH 7.0, 35.6 g/L Glu, 8 g/L Cel, 16.5 g/L Xyl.	40.1 9.8 -	[76]
*T. guehoae* UCDFST 6059	Corn stover hydrolysate. Bioreactor, 30 °C, pH 5.2, 1 vvm, 25% DO, 97.47 g/L Glu, 58.02 g/L Xyl, 3.51 g/L Gal, 8.56 g/L Ara, 0.24 g/L Fruc. Supplementation with 2 g/L YE, 4 g/L Pep and 1.6 g/L YNB.	48.3 14.2 0.120	[62]
*T. oleaginosus* ATCC 20509	Sorghum stalk hydrolysate. Flask, 25 °C, 200 rpm, 51% Glu, 30% Xyl, 2.9% Ara.	60.0 13.1 -	[61]
	Switchgrass hydrolysate. Flask, 25 °C, 200 rpm, 58% Glu, 26% Xyl.	58.0 12.3 -	
*Y. lipolytica* YB-392	Corn stover hydrolysate. Using 96-well plate, 25 °C, 400 rpm, pH 6.0, C/N 62:1, 59.3 g/L Glu, 36.3 g/L Xyl, 5.6 g/L Ara, 0.6 mM HMF, 0.09 mM Fur, 2.4 g/L AcA.	- 5.8 0.096	[60]
*Y. lipolytica* YB-437	Corn stover hydrolysate. Using 96-well plate, 25 °C, 400 rpm, pH 6.0, C/N 62:1, 59.3 g/L Glu, 36.3 g/L Xyl, 5.6 g/L Ara, 0.6 mM HMF, 0.09 mM Fur, 2.4 g/L AcA.	- 5.8 0.061	
*Y. lipolytica* Po1g	Sugarcane bagasse hydrolysate. Flask, 26 °C, 160 rpm, pH 6.5, 3.98 g/L Glu, 13.59 g/L Xyl, 2.78 g/L Ara.	58.5 6.7 0.070	[77]

Notes: The taxa displayed on the table refer to the original designation found in the corresponding articles. -, no data available. Lipid content = g of lipids/g of dry weight (%). Abbreviations: Acetic Acid (AcA); Arabinose (Ara); Butyric acid (ButA); Cellobiose (Cel); Corn Steep Solids (CSS); Dissolved oxygen (DO); Glucose (Glu); Fructose (Fruc); Furfural (Fur); Galactose (Gal); Mannose (Man); Peptone (Pep); References (Ref.); Xylose (Xyl); Yeast Extract (YE).

**Table 2:** Effect of the supplementation of cultivation media with different concentrations of furfural/HMF in lipid production by *Rhodotorula graminis*, *Lipomyces starkeyi*, *Rhodotorula glutinis*, *Rhodotorula toruloides* and *Trichosporon cutaneum*.

Species and Strain	Experimental Condition	Lipid Content (%) Lipid Titer (g/L) Lipid Productivity (g/Lh)	Ref.
*R. graminis* DBVPG 4620 (adapted strain)	Flask, CSL medium with HMF (0.4%)	- 1.8 -	[96]
*R. graminis* DBVPG 4620 (parental strain)		ND ND ND	
*R. graminis* DBVPG 4620	Flask, Medium B, control condition	43.0 5.52 0.061	[68]
	Flask, Medium B + Fur (1.5 g/L)	28.0 3.03 0.034	
	Flask, Medium B + HMF (1.5 g/L)	49.0 7.73 0.086	
*L. starkeyi* 2.1390	Flask, N-limited medium, control condition	37.2 2.29 0.021	[34]
	Flask, N-limited medium + 0.5 g/L Fur	30.3 1.64 -	
	Flask, N-limited medium + 0.5 g/L HMF	31.3 2.22 -	
*L. starkeyi* 2.1608	Flask, N-limited medium, control condition	21.8 2.04 0.097	
	Flask, N-limited medium + 0.5 g/L HMF	23.8 2.00 -	
*L. starkeyi* 2.1608	Flask, N-limited medium + 1.0 g/L HMF	24.6 2.08 -	[34]
*R. glutinis* 2.107	Flask, N-limited medium, control condition	13.0 0.52 0.042	
	Flask, N-limited medium + 0.5 g/L Fur	5.51 0.20 -	
	Flask, N-limited medium + 0.5 g/L HMF	11.0 0.56 -	
	Flask, N-limited medium + 1.0 g/L HMF	6.56 0.20 -	
	Flask, N-limited medium + 2.0 g/L HMF	8.19 0.24 -	
*R. glutinis* 2.704	Flask, N-limited medium, control condition	16.7 0.92 0.057	
	Flask, N-limited medium + 0.5 g/L HMF	6.43 0.34 0.023	
	Flask, N-limited medium + 1.0 g/L HMF	6.22 0.24 -	
	Flask, N-limited medium + 2.0 g/L HMF	4.49 0.14 -	
*R. toruloides* 2.1389	Flask, N-limited medium, control condition	39.3 1.67 0.044	
	Flask, N-limited medium + 0.5 g/L HMF	22.4 0.76 -	
	Flask, N-limited medium + 1.0 g/L HMF	16.5 0.40 -	
	Flask, N-limited medium + 2.0 g/L HMF	14.7 0.28 -	
*T. cutaneum* 2.1374	Flask, N-limited medium, control condition	39.8 1.09 0.011	
	Flask, N-limited medium+ 0.5 g/L Fur	42.5 1.25 -	
	Flask, N-limited medium + 1.0 g/L Fur	30.6 0.54 -	
*T. cutaneum* 2.1374	Flask, N-limited medium + 0.5 g/L HMF	46.8 0.83 -	[34]
	Flask, N-limited medium + 1.0 g/L HMF	44.2 1.14 -	
	Flask, N-limited medium + 2.0 g/L HMF	43.8 1.04 -	

Notes: The taxa displayed on the table refer to the original designation found in the corresponding articles. ND, not detected; -, no data available; Lipid content = g of produced lipids/g dry weight (%); Lipid titer = g of produced lipids/L of culture; Lipid productivity = g of produced lipids/L of culture per hour. Abbreviations: Corn steep liquor (CSL); Furfural (Fur); Nitrogen (N); References (Ref.).

**Table 3:** Lipid production by oleaginous yeasts (*Cryptococcus curvatus*, *Rhodotorula toruloides*, *Lipomyces starkeyi*, *Rhodotorula glutinis*, *Rhodotorula minuta*, *Rhodotorula mucilaginosa*, *Trichosporon cutaneum*, *Trichosporon fermentans* and *Yarrowia lipolytica*) using acetic acid as carbon source. The experimental conditions column also contains information on the culture method used: flasks, two-stage batch, sequential batch, fed-batch and semicontinuous fermentation.

Species and Strain	Experimental Condition	Lipid Content (%) Lipid Titer (g/L) Lipid Productivity (g/Lh)	Ref.
*R. toruloides* AS 2.1389	Flask, 20 g/L AcA, pH 6.0, C/N 200	48.2 - 0.025	[141]
	Flask, 4 g/L AcA, pH 6.0, C/N 230	15.2 - 0.009	
	Flask (*two-stage batch*), 1st step, 40 g/L Glu; 2nd step, 20 g/L AcA, pH 6.0, C/N 200	50.1 - 0.011	
	Flask (*two-stage batch*), 1st step, 40 g/L Glu; 2nd step, 5 g/L AcA, pH 6.0, C/N 200	13.7 - 0.002	
	Flask (*Sequencing batch*), 4 g/L AcA, pH 6.0, C/N 100	38.6 - 0.024	
*C. curvatus* ATCC 20509	Bioreactor (*fed-batch*), AcA (5 g/L), pH 7.0, C/N 300	53.0 25.0 -	[144]
	Flask, acetate assimilation medium [30 g/L AcA], pH 7.0, C/N 50	73.4 4.2 -	[33]
	Bioreactor, N-rich acetate medium, containing 5 g/L AcA, pH 7.0, C/N 1.76	56.7 0.8 0.030	
	Bioreactor, N-limited acetate medium containing 30 g/L AcA, pH 7.0, C/N 33.5	66.4 3.4 0.033	
*L. starkeyi * AS 2.1560	Flask, acetate assimilation medium [30 g/L AcA], pH 7.0, C/N 50	17.1 0.6 -	
*R. glutinis * AS 2.107	Flask, acetate assimilation medium [30 g/L AcA], pH 7.0, C/N 50	27.0 0.7 -	[33]
*R. minuta * AS 2.277		30.2 0.5 -	
*R. mucilaginosa* AS 2.1515		21.8 0.6 -	
*R. toruloides* ATCC 10788		33.0 0.4 -	
*R. toruloides* Y4		54.9 1.5 -	
*T. cutaneum* AS 2.571		58.5 4.4 -	
*T. fermentans* CICC 1368		55.4 3.8 -	
*Y. lipolytica* AS 2.1398		12.2 0.5 -	
*Y. lipolytica* MUCL 28849	Bioreactor (fed-batch), 3x4 g/L AcA, pH 5.6, C/N 50	30.8 1.8 -	[145]
	Bioreactor (two-stage fed-batch), 1st step, 40 g/L Glu; 2nd step, 5 g C/L AcA, pH 5.6, C/N 50	40.7 12.4 0.160	
	Bioreactor (two-stage fed-batch), 1st step, 40 g/L Gly; 2nd step, 5g C/L AcA, pH 5.6, C/N 50	38.4 15.7 0.330	
*Y. lipolytica* MTYL065	Bioreactor (semicontinuous fermentation), 3% AcA feed, pH 7.0, C/N 32	52.6 10.0 0.070	[87]
	Bioreactor (semicontinuous fermentation), joint feed of 3% acetic acid and acetate, C/N 13.2 (first 72 h) C/N 102 afterward.	56.9 33.0 0.230	
	Bioreactor (semicontinuous fermentation), optimised carbon and nitrogen feed.	59.2 115.0 0.800	
	Bioreactor (semicontinuous fermentation), optimised carbon and nitrogen feed.	59.2 115.0 0.800	

Notes: The taxa displayed on the table refer to the original designation found in the corresponding articles. -, no data available. Lipid content: g lipids/g dry weight (%); Lipid concentration: g of produced lipid/L of culture; Lipid yield: g of produced lipids/L of culture per hour. Abbreviations: Acetic acid (AcA); Glucose (Glu); Glycerol (Gly); Nitrogen (N); References (Ref.).

**Table 4:** Lipid production by *Cryptococcus curvatus*, *Naganishia uzbekistanensis*, *Rhodotorula glutinis*, *Rhodotorula kratochvilovae*, *Rhodotorula toruloides*, *Trichosporon fermentans*, *Trichosporon oleaginosus* and *Yarrowia lipolytica* using crude glycerol as C-source.

Species and Strain	Experimental Condition	Lipid Content (%) Lipid Titer (g/L) Lipid Productivity (g/Lh)	Ref.
*Y. lipolytica * SKY7	Bioreactor (fed-batch), 20 g/L suspended solids of washed sludge fortified with crude Gly (10.08 g/L FFA with 5.78 g/L Gly), 28 °C, 400–600 rpm, pH 6.0.	- 31.4 -	[148]
*Y.lipolytica* FMCC Y_73_	Flask, 40 g/L Gly (crude Gly purity = 90%), supplemented with 2.0 g/L Pep and 1.0 g/L YE. 29 °C, 190 rev. min^−1^, pH 6.0.	16.9 1.2 -	[149]
*Y.lipolytica* FMCC Y_74_		10.7 0.8 -	
*Y.lipolytica* FMCC Y_75_		19.1 1.3 -	
*Rhodotorula* sp. FMCC Y_78_		11.3 1.0 -	
*Rhodotorula* sp. FMCC Y_76_		18.7 1.7 -	
*R. glutinis* NRRL YB-252		19.3 2.1 -	
*C. curvatus* NRRL Y-1511		8.4 1.3 -	
*N. uzbekistanensis* FMCC Y_72_		34.4 1.1 -	
*R. kratochvilovae * FMCC Y_70_		19.8 1.7 -	
*R. kratochvilovae * FMCC Y_71_		16.7 1.5 -	
*Debaryomyces sp.* FMCC Y_68_		29.9 2.0 -	
*Debaryomyces sp.* FMCC Y_69_	Flask, 40 g/L Gly (crude Gly purity = 90%), supplemented with 2.0 g/L Pep and 1.0 g/L YE. 29 °C, 190 rev. min^−1^, pH 6.0.	22.4 1.7 -	[149]
*Debaryomyces sp.* FMCC Y_68_	Flask, 80 g/L Gly (crude Gly purity = 90%), supplemented with 2.0 g/L Pep and 1.0 g/L YE. 29 °C, 190 rev. min^−1^, pH 6.0.	16.9 2.1 -	
*Debaryomyces sp.* FMCC Y_69_		38.9 2.3 -	
*R. glutinis* NRRL YB-252		38.2 7.2 -	
*C. curvatus* NRRL Y-1511		23.9 4.5 -	
*N. uzbekistanensis* FMCC Y_72_	Flask, 55 g/L Gly (crude Gly purity = 90%), supplemented with 2.0 g/L Pep and 1.0 g/L YE. 29 °C, 150–450 rev. min^−1^, pH 6.0, 2 vvm.	31.1 3.3 -	
*T. oleaginosus* ATCC 20905	Bioreactor (*fed-batch*), 6.07 g/L Gly (from a crude Gly with a purity of 15.05%), non-sterilised conditions, 30 °C, 300–500 rpm, pH 5.0, DO > 35% (*v*/*v*).	48.1 20.8 -	[150]
	Bioreactor (*fed-batch*), 6.22 g/L Gly (from a crude Gly with a purity of 15.05%) + 10.08 g/L FFA 30 °C, pH 5.0, DO > 30% (*v*/*v*).	54.5 35.8 -	[151]
	Bioreactor (*batch*), 10.28 g C/L (crude Gly purity = 13.24%), C/N 20, 30 °C, 300–500 rpm, pH 5.0, DO > 35%.	23.0 3.1 0.05	[152]
	Bioreactor (*batch*), 15.30 g C/L (crude Gly purity = 13.24%), C/N 30, 30 °C, 300–500 rpm, pH 5.0, DO > 35%.	47.5 11.3 0.21	
	Bioreactor (*batch*), 22.84 g Carbon/L (crude Gly purity = 13.24%) C/N 45, 30 °C, 300–500 rpm, pH 5.0, DO > 35%.	49.0 12.1 0.22	
	Bioreactor (*batch*), 29.69 g Carbon/L (crude Gly purity = 13.24%) C/N 60, 30 °C, 300–500 rpm, pH 5.0, DO > 35%.	52.0 10.0 0.18	
	Bioreactor (*fed*-*batch*), ~46.26 g Carbon/L (crude Gly purity = 13.24%) C/N 45, 30 °C, 300–500 rpm, pH 5.0, DO > 35%.	49.9 21.9 0.42	
*T. cutaneum * AS 2.0571	Flask, 70 g/L crude Gly (75.1% purity) C/N 60, 30 °C, 200 rpm.	32.2 5.6 -	[153]
*T. fermentans * CICC 1368	Flask, 50 g/L crude Gly (75.1% purity) C/N 60, 25 °C, 160 rpm.	32.4 5.2 -	[153]
*Y. lipolytica* A101	Flask, 50 g/L crude Gly (purity of 80%, from soap production). Supplementation with YNB and (NH_4_)_2_SO_4_. C/N 100, 28 °C, 240 rpm, pH 6.0.	24.9 1.7 -	[154]
	Flask, 50 g/L crude Gly (purity of 80%, from biodiesel). Supplementation with YNB and (NH_4_)_2_SO_4_. C/N 100, 28 °C, 240 rpm, pH 6.0.	24.3 0.9 -	
	Flask, 50 g/L crude Gly (purity of 42%, from stearin production). Supplementation with YNB and (NH_4_)_2_SO_4_. C/N 100, 28 °C, 240 rpm, pH 6.0.	28.0 0.7 -	
*R. toruloides* 32489	Flask, crude Gly concentration equivalent to a carbon weight of 20 g/L Glu (crude Gly purity = 49%). Supplementation with 2 g/L Pep, 0.5 g/L (NH_4_)_2_SO_4_, 1 g/L K_3_PO_4_ and 0.5 g/L MgSO_4_. C/N 60, 30 °C, 200 rpm, pH 7.0.	41.8 6.2 -	[155]
*R. glutinis * TISTR 5159	Bioreactor (*fed-batch*), 9.5% crude Gly (50% purity) C/N 85, 30 °C, 100 rpm, pH 6.0, 2 vvm.	60.7 6.05 -	[156]
*C. curvatus * ATCC 20509	Bioreactor (*fed-batch*), 100 g/L Gly (crude Gly purity = 91%) + sunflower meal hydrolysate, 28 °C, 200–700 rpm, pH 6.0, 1vvm.	47.1 17.9 0.09	[157]
	Bioreactor (*fed-batch*), 100 g/L Gly (crude Gly purity = 91%) + pretreated sunflower meal hydrolysate, 28 °C, 200–700 rpm, pH 6.0, 1vvm.	52.9 18.3 0.11	
*R. toruloides* DSM 4444	Bioreactor (*fed-batch*), 50 g/L Gly (crude Gly purity = 91%) + sunflower meal hydrolysate, 28 °C, 200–700 rpm, pH 6.0, 1vvm.	37.8 18.1 0.14	
	Bioreactor (*fed-batch*), 50 g/L Gly (crude Gly purity = 91%) + pretreated sunflower meal hydrolysate, 28 °C, 200–700 rpm, pH 6.0, 1vvm.	51.3 19.2 0.17	
*Y. lipolytica* ATCC 20460	Flask, 343 mM Gly (from crude Gly purity between 78–86%) 30 °C, 125 rpm.	11.6 - -	[158]
*R. toruloides* Y4	Flask, 20 g/L Gly (from a crude Gly containing 50 g/L Gly) 30 °C, 200 rpm, pH 5.5.	21.6 2.5 -	[159]
	Flask, 50 g/L Gly (from a crude Gly containing 50 g/L Gly) 30 °C, 200 rpm, pH 5.5.	35.4 6.9 -	
	Flask, 100 g/L Gly (from a crude Gly containing 50 g/L Gly) 30 °C, 200 rpm, pH 5.5.	42.5 8.6 -	
	Flask, 150 g/L Gly (from a crude Gly containing 50 g/L Gly) 30 °C, 200 rpm, pH 5.5.	40.4 6.6 -	
	Flask, 200 g/L Gly (from a crude Gly containing 50 g/L Gly) 30 °C, 200 rpm, pH 5.5.	41.5 5.6 -	
*C. curvatus * ATCC 20509	Bioreactor (*fed-batch*), 25.8 g/L glycerol (crude Gly purity = 48.7%). Supplementation with NH_4_Cl. C/N 30, 28 °C, pH 5.5.	44.6 13.92 0.05	[160]
*C. curvatus * ATCC 20509	Bioreactor (*fed-batch*), 32 g/L Gly (crude glycerol purity = 48.7%). Supplementation with NH_4_Cl. C/N 30, 28 °C, pH 5.5.	52.9 17.40 0.06	[160]

Notes: The taxa displayed on the table refer to the original designation found in the corresponding articles. -, no data available; Lipid content: g lipids/g dry weight (%); Lipid concentration: g of produced lipid/L of culture; Lipid yield: g of produced lipids/L of culture per hour. Abbreviations: Glycerol (Gly); References (Ref).

**Table 5:** Lipid production by oleaginous yeasts (*Cryptococcus albidus*, *Cryptococcus curvatus*, and *Yarrowia lipolytica*) using a mixture of VFAs as carbon source. The experimental conditions column also contains information on the culture method used: flasks, two-stage batch, sequential batch and fed-batch.

Species and Strain	Experimental Condition	Lipid Content (%) Lipid Titer (g/L) Lipid Productivity (g/Lh)	Ref.
*C. curvatus* ATCC 20509	Bioreactor (fed-batch), VFAs, mainly acetate and butyrate (12 + 4 g/L), pH 7.0, C/N increased to 15 when [VFAs] reached 2 g/L.	42.0 - -	[171]
*C. curvatus * MUCL 29819	Flask (Sequencing batch), 3.35 g/L VFAs from activated sludge.	39.6 - 0.03	[172]
*C. albidus* ATCC 10672	Bioreactor, AcA: ProA: ButA (5:1:4), pH 6.0 COD/N ratio 25:1.	28.3 0.32 -	[173]
*C. curvatus* ATCC 20509	Bioreactor (repeated batch), VFAs (9.27 g/L), pH 7.0.	61.0 1.36 -	[174]
*Y. lipolytica* MUCL 28849	Bioreactor (two-stage fed-batch), 1st step, 40 g/L Glucose; 2nd step, 5g C/L [AcA:ProA:ButA] (3:1:1), pH 5.6, C/N 50.	40.2 16.50 0.33	[145]
	Bioreactor (two-stage fed-batch), 1st step, 40 g/L Glycerol; 2nd step, 5g C/L [AcA:ProA:ButA] (3:1:1), pH 5.6, C/N 50.	34.6 14.19 0.28	
*C. albidus var. albidus* ATCC 10672	Flask, 2 g/L [AcA:ProA:ButA] (4:3:3), pH 6.0.	19.8 0.13 -	[170]
	Flask, 2 g/L [AcA:ProA:ButA] (8:1:1), pH 6.0.	27.8 0.33 -	
	Flask, 2 g/L [AcA:ProA:ButA] 7:2:1, pH 6.0.	26.1 0.29 -	
	Flask, 2 g/L [AcA:ProA:ButA] 6:1:3, pH 6.0.	27.0 0.31 -	
	Flask, 5 g/L [AcA:ProA:ButA] 6:1:3, pH 6.0.	24.9 0.64 -	
	Flask, 8 g/L [AcA:ProA:ButA] 6:1:3, pH 6.0.	11.9 0.09 -	

Notes: The taxa displayed on the table refer to the original designation found in the corresponding articles. -, no data available; Lipid content: g lipids/g dry weight (%); Lipid concentration: g of produced lipid/L of culture; Lipid yield: g of produced lipids/L of culture per hour. Abbreviations: Acetic acid:propionic acid:butyric acid (AcA:ProA:ButA).

## Data Availability

Not applicable.
